# Perceived barriers and facilitators for model-informed dosing in pregnancy: a qualitative study across healthcare practitioners and pregnant women

**DOI:** 10.1186/s12916-024-03450-8

**Published:** 2024-06-18

**Authors:** Charlotte Koldeweij, Mirèse Kleuskens, Carlijn Litjens, Bryony Dean Franklin, Hubertina C. J. Scheepers, Saskia N. de Wildt

**Affiliations:** 1 Division of Pharmacology and Toxicology, Department of Pharmacy, Radboud University Medical Center, Nijmegen, The Netherlands; 2 Netherlands Pharmacovigilance Centre Lareb, ‘s-Hertogenbosch, The Netherlands; 3 Centre for Medication Safety and Service Quality, Imperial College Healthcare NHS Trust, London, UK; 4 Department of Practice and Policy, UCL School of Pharmacy, London, UK; 5 Department of Obstetrics and Gynaecology, Maastricht University Medical Centre, Maastricht, The Netherlands; 6 Grow, School for Oncology and Reproduction, Maastricht, The Netherlands; 7 Department of Pediatric and Neonatal Intensive Care, Erasmus MC-Sophia Children’s Hospital, Rotterdam, The Netherlands

**Keywords:** Dose, Antenatal, Pregnancy, Pharmacokinetic modelling, Shared decision-making

## Abstract

**Background:**

Most women use medication during pregnancy. Pregnancy-induced changes in physiology may require antenatal dose alterations. Yet, evidence-based doses in pregnancy are missing. Given historically limited data, pharmacokinetic models may inform pregnancy-adjusted doses. However, implementing model-informed doses in clinical practice requires support from relevant stakeholders.

**Purpose:**

To explore the perceived barriers and facilitators for model-informed antenatal doses among healthcare practitioners (HCPs) and pregnant women.

**Methods:**

Online focus groups and interviews were held among healthcare practitioners (HCPs) and pregnant women from eight countries across Europe, Africa and Asia. Purposive sampling was used to identify pregnant women plus HCPs across various specialties prescribing or providing advice on medication to pregnant women. Perceived barriers and facilitators for implementing model-informed doses in pregnancy were identified and categorised using a hybrid thematic analysis.

**Results:**

Fifty HCPs and 11 pregnant women participated in 12 focus groups and 16 interviews between January 2022 and March 2023. HCPs worked in the Netherlands (*n* = 32), the UK (*n* = 7), South Africa (*n* = 5), Uganda (*n* = 4), Kenya, Cameroon, India and Vietnam (*n* = 1 each). All pregnant women resided in the Netherlands. Barriers and facilitators identified by HCPs spanned 14 categories across four domains whereas pregnant women described barriers and facilitators spanning nine categories within the same domains. Most participants found current antenatal dosing information inadequate and regarded model-informed doses in pregnancy as a valuable and for some, much-needed addition to antenatal care. Although willingness-to-follow model-informed antenatal doses was high across both groups, several barriers for implementation were identified. HCPs underlined the need for transparent model validation and endorsement of the methodology by recognised institutions. Foetal safety was deemed a critical knowledge gap by both groups. HCPs’ information needs and preferred features for model-informed doses in pregnancy varied. Several pregnant women expressed a desire to access information and partake in decisions on antenatal dosing.

**Conclusions:**

Given the perceived limitations of current pharmacotherapy for pregnant women and foetuses, model-informed dosing in pregnancy was seen as a promising means to enhance antenatal care by pregnant women and healthcare practitioners.

**Supplementary Information:**

The online version contains supplementary material available at 10.1186/s12916-024-03450-8.

## Background

Over eighty per cent of women use medications during pregnancy [1]. While the last decades have been marked by efforts to expand data on the foetal safety of medications, most of which are used off-label in pregnancy [2], the lack of pharmacokinetic data in pregnancy has gained attention with the growing knowledge that physiological changes in pregnant women’s bodies may alter the maternal and foetal disposition of medications [3]. In turn, pharmacokinetic changes in pregnancy may affect medication efficacy and safety, in some cases warranting dosing alterations [3, 4].

The lack of evidence-based doses in pregnancy represents a large unmet need for pregnant women and their unborn children. Pregnant women may receive standard doses normally prescribed to non-pregnant adults, or, in some cases, empirically adjusted doses by their healthcare practitioner (HCP). The lack of pregnancy-adjusted doses may lead to toxicity or suboptimal efficacy of treatment, creating unnecessary risks for mother and child [4].

Recently, model-informed dosing (MID), or the use of pharmacokinetic models simulating medication behaviour in the body, has emerged as an innovative approach to tailor medication doses for diverse patient groups [5]. In a context where the enrolment of pregnant women in clinical studies remains limited by ethical, practical and regulatory concerns [6, 7], MID provides an alternative means to generate evidence on adequate antenatal doses [5, 8]. Both population-based (pop-PK) and physiologically-based (PBPK) pharmacokinetic models can be used to simulate maternal and foetal drug exposures based on limited clinical data [8]. PBPK models incorporate pregnancy-induced changes in medication absorption, distribution, metabolism and elimination to investigate adequate doses throughout pregnancy [5]. Pop-PK models describe the pharmacokinetics of a medication in a population drawing on concentration samples and co-variate analysis to account for individual variability [8]. By comparing the predicted plasma concentrations for various doses with reference concentrations for safety and efficacy, the most fitting dosing regimen can be chosen [8]. Such models can thus be leveraged to examine potential adjustments in antenatal doses and help optimise maternofoetal therapies [8].

MID could be used alongside clinical data to inform evidence-based doses in pregnancy. This prospect is currently explored as part of project MADAM (Model-Adjusted Doses for All Mothers), an international effort seeking to establish proof-of-concept for a model-informed pregnancy formulary (MIPF). The envisaged MIPF will be an openly accessible resource on maternal and foetal medication doses based on in-depth evidence reviews, including simulated data, by multidisciplinary experts. This approach, while promising, represents a departure from current antenatal dosing practices. Alongside scientific challenges in model development and validation, previously identified barriers for MID implementation in other patient groups include sociocultural factors such as varying expertise and language among modellers and clinical users, potentially affecting knowledge transfer [9, 10]. Successful implementation of a MIPF thus requires a better understanding of the barriers and facilitators perceived by stakeholders involved in making antenatal dosing decisions. The current study aims to explore the perceptions of stakeholders regarding the feasibility and acceptability of model-informed dosing in pregnancy as part of an effort to promote the adoption of a MIPF in routine antenatal care.

## Methods

### Study setting

Focus groups and interviews were conducted online with participants primarily from the Netherlands and Belgium, as well as six other countries across Europe, Africa and Asia.

### Study design

Online focus groups and interviews constituted the initial step of a cross-sectional qualitative study on the perceived barriers and facilitators for the implementation of a MIPF among HCPs and pregnant women.

### Definitions

A model-informed pregnancy formulary (MIPF) was defined as a central resource comprising specifically researched medication dose recommendations for pregnant women and foetuses, in part drawing on modelling and simulations derived from PBPK or pop-PK models. The latter were referred to as model-informed doses.

### Participants

#### Eligibility

We aimed to recruit participants representing the two main groups of stakeholders involved in shared decision-making on antenatal medication dosing: HCPs and pregnant women. HCPs comprised registered prescribers across various medical specialties including gynaecologists-obstetricians, obstetric physicians, internists, psychiatrists, neurologists, anaesthetists and general practitioners as well as non-prescribers including pharmacists and midwives. Eligible participants included both experienced HCPs and those undergoing training following their qualifying degree. Women were eligible if they were currently pregnant or had been pregnant in the last three years; medication use during pregnancy was not required. Participants had to have conversational mastery of either Dutch or English. There were no geographic restrictions on participation.

#### Recruitment

Purposive convenience sampling was used for recruitment. A diverse group of HCP participants was sought, including both individuals with knowledge of pharmacokinetic models or clinical pharmacology and clinicians without such expertise. HCPs were recruited through mailing lists or websites from professional societies representing a broad range of healthcare professionals including various medical specialists involved in caring for pregnant women, pharmacists and pharmacologists. In addition, individual HCPs were contacted based on their relevant expertise including perinatology or clinical pharmacology. Pregnant women were recruited through general pregnancy websites and social media, along with flyers in consultation rooms across various care settings. The focus groups were also advertised on the website of the Dutch Teratology Information Service (Pharmacovigilance Centre Lareb Moeders van Morgen). Interested individuals received an email with information on the goals of the focus group and were asked for demographic information and their informed consent for participation and storage of anonymised transcripts of the focus groups. No financial incentives were offered for participation.

### Data collection

Data collection took place between January 2022 and March 2023. All data was collected online. HCPs and pregnant women took part in separate focus groups. Pregnant women with a medical profession were eligible to take part and were allocated to the focus group of their choice (either HCPs or pregnant women). This approach was adopted to avoid placing restrictions on their participation that might make them less comfortable sharing their experiences during focus groups. Participants were further assigned to a focus group based on their language and availability, and for HCPs, their country of work and specialty. Similarity in background was sought to reduce barriers to communication. Focus groups that comprised three to six participants were aimed for. When interested individuals were not available for a focus group, they were given the alternative of participating in an interview that could have one or two participants. The focus groups and interviews occurred through videoconferencing (Microsoft Teams version 1.6.00.17554) and were recorded and automatically transcribed. The focus groups and interviews were facilitated by a skilled moderator with expertise in clinical medicine and qualitative research and proficiency in English and Dutch (CK). An observer (CL or MK) was present for technical support. The conversations followed a semi-structured approach, drawing on topic guides (Additional file 1) developed by the multidisciplinary research team based on their expertise and an exploratory literature search on MID and implementation science. Distinct topic guides were developed for HCPs and pregnant women to ensure knowledge and experience fit as well as to gain a sufficient understanding of antenatal pharmacological care in settings outside the Netherlands where the moderator was trained and worked as a clinician. Covered themes included participants’ dosing practices and preferences, information needs, perceptions of MID, preferred features for a MIPF and expectations regarding the implementation process, alongside setting-specific questions on the organisation of antenatal pharmacological care. Participants were given a presentation on MID in pregnancy and the preliminary features of the envisioned MIPF, as previously defined, during their focus group or interview**.**

### Outcome measures

The perceived barriers and facilitators for setting up and disseminating the use of a MIPF formed the core outcomes of this research.

### Patient and public involvement

The topic guides and presentations were refined through user-testing as part of two preliminary focus groups, one with HCPs and the other with pregnant women.

### Ethics and reporting

The study protocol was assessed by the Medical Ethics committee of Radboud University Medical Centre (2021–13287) and was not subject to the Medical Research Involving Human Subjects Act. This paper is reported following the EQUATOR-approved Standards for Reporting Qualitative Research Checklist (Additional file 2) [11].

### Data analysis

Data analysis drew on critical realist epistemology and an interpretivist approach [12]. The transcripts were coded manually by two researchers (CK and MK). For the first three transcripts, each researcher independently identified free text corresponding to barriers and facilitators — namely, factors that participants deemed likely to affect the adoption of the envisioned MIPF in routine antenatal care. Free text selections were compared among researchers, and barriers and facilitators grouped thematically through a hybrid inductive and deductive analysis. Recurring themes were clustered into categories and subcategories, with accompanying free text quotes providing illustration. In addition, emerging categories and subcategories were deductively coded against a published conceptual framework by van Sluisveld et al., [13] itself based on three frameworks from implementation science [14–16]. Sluisveld et al.’s framework comprised several domains influencing the clinical implementation of evidence, including intervention characteristics, provider and patient characteristics, and implementation characteristics. Certain domains and categories of Sluisveld et al.’s framework were merged or adapted to fit the data. Additionally, the framework was enriched with categories and subcategories identified through inductive analysis of additional transcripts, conducted by individual researchers. Discrepancies between researchers were solved through consensus. Thematic saturation was assessed through consensus within the research team to ascertain when an adequate sample size had been reached [17]. Thematically organised barriers and facilitators were listed in tables. Barriers and facilitators identified by HCPs from countries other than the Netherlands and the UK were specifically labelled. Likewise, barriers and facilitators that were exclusively outlined by pregnant women with a medical background were identified.

### Reflexivity statement

CK is a medical doctor and a social scientist, and MK is a biomedical student. CL is a toxicologist. BDF is a pharmacist and professor of medication safety. LS is a gynaecologist and associate professor in obstetrics. SDW is a paediatric intensivist and a professor of clinical pharmacology. All authors, apart from BDF who worked in the UK, were primarily based in the Netherlands. CK, LS and BDF have prior experience in conducting and analysing qualitative studies in the clinical environment. The authors anticipated the presence of barriers and facilitators for the implementation of a MIPF but strived to prevent preconceived notions from biasing data interpretation. The team acknowledged the potential influence of their backgrounds on study design, analysis, and interpretation. They sought to maintain a reflexive stance throughout to minimise the risk of bias or assumptions affecting the analysis.

### Data credibility and reliability

Transcript content was cross-checked against the video recordings for accuracy (MK, CL, CK and CD). In addition, the credibility of the analysis was promoted through thorough familiarisation of researchers with the data including notetaking and debriefing during data collection, and double reading and debriefings as part of data analysis. Partial reliance on an existing framework for coding consistency further enhanced the trustworthiness of the analysis. Inter-coder reliability was tested on the first three transcripts analysed, while thematic saturation was used to ensure a comprehensive understanding of the data.

### Data management and storage

Video recordings were deleted after transcript verification. Transcripts were anonymised by assigning codes to participants and removing identifying details. Transcripts were password-protected and accessible to authorised data coders only.

## Results

### Demographic characteristics

Twelve focus groups, with a median of four participants, and 16 interviews, comprising 14 individual interviews and two interviews with two participants each, were carried out. Sixteen focus groups and interviews were conducted in Dutch, and twelve in English. In total, 50 HCPs and 11 pregnant women participated. HCP participants worked in the Netherlands (*n* = 32), the UK (*n* = 7), South Africa (*n* = 5), Uganda (*n* = 4), Kenya, Cameroon, India and Vietnam (*n* = 1 each). HCPs across a wide range of specialties participated (Table 1). The majority of HCPs worked in an academic hospital. All pregnant women resided in the Netherlands. Seven of 11 pregnant women held a university degree. Five were medical professionals (four doctors and one nurse) and three used long-term medication during their pregnancy. More detailed information on the composition of the focus groups and interviews is outlined in Additional file 3.

**Table 1:** Baseline characteristics of participants

Healthcare practitioners (*n* = 50)	
**Country/region of work**	
Cameroon	1
India^a^	1
Kenya	1
Netherlands	32
South Africa	5
Uganda	4
UK^a^	7
Vietnam^a^	1
**Gender**	
Female	39
Male	11
**Specialty**	
Community pharmacist	3
General practitioner	5
Gynaecologist-obstetrician	11(2)^b^
Hospital pharmacist or clinical pharmacologist^c^	7
Internist or general physician	6(2)
Midwife	4
Obstetric physician	3
Other medical specialist (anaesthesia, gastro-enterology, neurology, psychiatry)	7
Research clinician	2
Other	2
**Organisation**	
Academic hospital	28^d^
General hospital	13
Community care	10
**Pregnant women (*n* = 11)**	
**Pregnancy**	
Currently pregnant	5
Recently pregnant	6
**Place of residence in the Netherlands**	
Middle	4
North-west	1
South-east	3
South-west	3
**Highest educational degree**	
High school	1
University of applied science	3
University	7
**Medical profession**	
Yes	5
No	6
**Long-term medication use during pregnancy**	
Yes	3
No	8

^a^One UK-based physician also worked in Vietnam and India

^b^Numbers in parentheses were used to describe medical specialists who were additionally trained as clinical pharmacologists

^c^Clinical pharmacologists were only counted in this category if not simultaneously working in another specialty

^d^One healthcare practitioner worked in both an academic and a general hospital

### Thematic analysis

#### HCPs

**Fig. 1:**
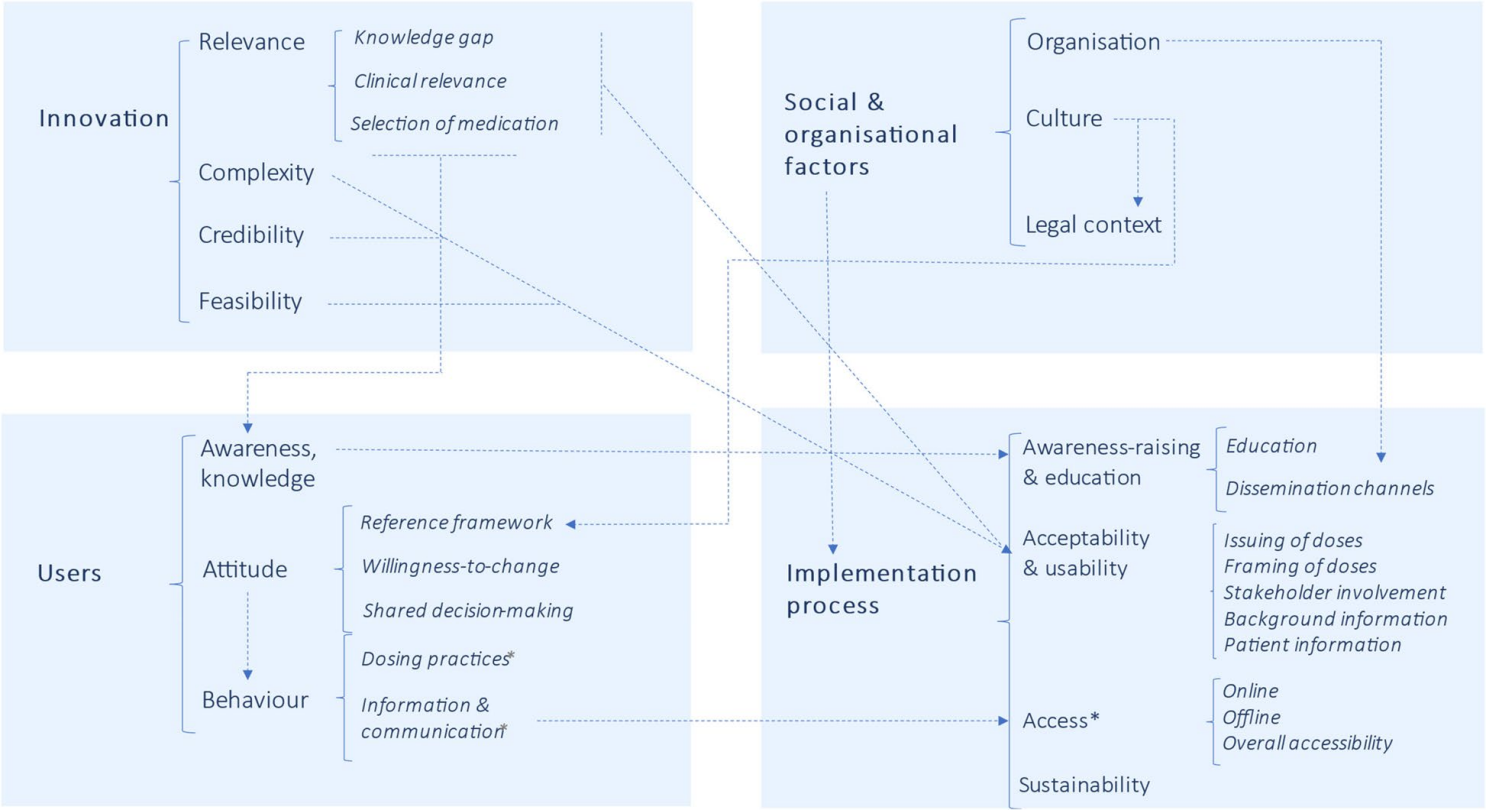
Perceived barriers and facilitators for the implementation of model-informed dosing in pregnancy according to healthcare practitioners. The dotted arrows depict some of the relationships between domains, categories and subcategories of themes

As shown in Table 2, barriers and facilitators identified by HCPs fell under 14 categories across four domains: the innovation, users, socio-organisational factors and the implementation process. Seven categories from Sluisveld et al.’s framework were removed, fifteen were adapted or merged, and two were added (Additional file 4). Overarching themes are described below with further quotes in Additional file 5. Relationships between domains and categories are depicted in Fig. 1.

**Table 2:** Perceived barriers and facilitators for the implementation of model-informed dosing in pregnancy according to healthcare practitioners

Category	Subcategory	Barrier	Facilitator
**Innovation**			
**Relevance**	Knowledge gap	* **The lack of evidence on foetal safety is a critical knowledge gap**	* MIDs could help address the lack of evidence-based doses in pregnancy
	Clinical relevance	* **Models do not provide information on foetal safety** * Employed models do not cover post-partum and lactation periods * Models do not capture pregnancy effects on pharmacodynamics * **Need for individualised doses taking into account a PW’s (patho)physiological features (e.g. BMI, comorbidities)**	* **MIDs could be integrated into a complete and up-to-date resource on medication in pregnancy, which is currently missing** * **MIDs can significantly improve the quality of antenatal care** * Models generate data without exposing PW and foetuses to harm * Models give information on maternal physiology and foetal exposure * *Models can be used to explore genetic variation in pharmacokinetics *
	Selection of medication		* Include most frequently used medications in pregnancy (across specialties) * Include medications for treating top global causes of mortality and morbidity
**Complexity**		* A good understanding of models requires training and expertise * Can models capture the complex and changing physiology of pregnant women and their foetuses?	
**Credibility**		* HCPs are only willing to follow MIDs if model predictions have been validated against clinical data * Some HCPs require clinical experience with MIDs	* Transparency on model assumptions can enhance model credibility * Specify and share standards for model validation * *Verify the accuracy of model predictions in (e.g. genetically diverse) subgroups of PW* * Verify MIDs a posteriori against data from routine medication use (e.g. TDM)
**Feasibility**		* Building a comprehensive MIPF is labour-intensive * Limited data for model parametrisation and verification * Some medications lack a therapeutic range	* Collaborate with other research groups for model development * Leverage existing*, if possible locally relevant*, pharmacokinetic data * Prioritise medications with known therapeutic target (e.g. antibiotics)
**Users**			
**Awareness, knowledge**		* Most prescribers have limited knowledge of pharmacokinetics * Few HCPs know about pharmacokinetic models * Awareness of altered pharmacokinetics in pregnancy varies	* Some HCPs already apply complex dosing strategies, including in pregnancy
**Attitude**	Reference framework	* **HCPs often prioritise foetal safety over maternal efficacy** * **HCPs are conservative when prescribing or dosing medications in pregnancy out of concern for foetal safety** * **PW primarily care about foetal safety**	* Mostly positive stance on the potential applications of modelling across various domains including healthcare * Information shared by national TISes is perceived as trustworthy
	Willingness-to-change	* HCPs require varying levels of access to evidence from modelling to be willing to follow MIDs	* **Many HCPs see MIDs as the best available evidence for antenatal dosing**
	Shared decision-making	* **While PW must be better informed on the risks and benefits of medication use, mixed opinions on whether this applies to dosing;** different PW have different information needs * Shared decision-making during pregnancy is important, however dosing is a complex topic	* Shared decision-making is not required for dosing * Greater medication efficacy could enhance PW’s therapy adherence
**Behaviour**	Dosing practices	* HCPs mostly follow doses recommended for the general adult populations, including for PW * TDM is used for a minority of medications	* Some HCPs adjust medication doses in pregnancy on an ad hoc basis (based on experience and/or a PW’s symptoms)
	Information and communication	* **HCPs consult various resources on antenatal medication** * Limited time to inform PW *(especially in lower resource settings)*	* Some HCPs discuss doses with PW, especially in regards to side effects
**Social and organisational factors**			
**Organisation**		* Pharmacological care in pregnancy is interdisciplinary * *Medication and formulation availability varies per country*	* Professional bodies and national guideline committees define best practice (hence MIDs they endorse can be applied (inter)nationally)
**Culture**		* **Society values foetal safety over maternal health** * Decision-making in pregnancy involves high uncertainty * HCPs value their professional autonomy	* High value assigned to evidence-based care outlined in clinical guidance * High level of trust in professional bodies and guidelines committees
**Legal context**		* Liability from fetal adverse events is an important concern	
**Implementation process**			
**Awareness-raising and education**	Education	* Varying levels of medical training of HCPs depending on geographic setting (e.g. community health workers)	* Develop proof-of-concept dose recommendations for selected medications * Integrate trainings on pharmacokinetics and MIDs in medical school curriculums * Organise accredited trainings on pharmacokinetics and MID in pregnancy
	Dissemination channels	* High diversity of targeted MIPF end-users	* Introduce MIPF at congresses and in journals of relevant specialties * Include doses in (inter)national clinical guidelines and formularies (e.g. *WHO*) * Draw on local champions for the clinical implementation of MIPF doses
**Acceptability and usability**	Issuing of doses	* Liability for pregnancy-adjusted doses must be clarified	* Obtain approval of doses by multidisciplinary experts and targeted end-users * Seek endorsement of MIPF by professional associations, pharmaceutical regulatory agencies, and (inter)national guideline committees
	Framing of doses		* Develop clear and actionable dose recommendations (e.g. per trimester) * Indicate the level of certainty of doses (e.g. grade of evidence, tailored wording) * **Outline information to help HCPs individualise and/or monitor the therapeutic and/or toxic effects of recommended doses**
	Stakeholder involvement		* Involve end-users (HCPs and patients) in MIPF design * Ensure broad representation of stakeholders in the working committee
	Background information		* Provide access to underlying evidence and/or risk–benefit analysis * Share information on physiological changes and pharmacokinetics in pregnancy * Share information on foetal exposure to individual medications
	Patient information		* Most HCPs see added value of concise patient information on MIDs (whilst avoiding information overload)
**Access**	Online	* **Mixed views on preferred home for MIPF**: existing (e.g. national formulary, TIS) or stand-alone website * Level of awareness and use of Dutch TIS website varies across medical specialties	* Most HCPs prefer a single online resource on dosing in pregnancy * Ensure that online dosing information is layered and easy to navigate * Link MIPF to other websites e.g. formularies and electronic health records * Make MIPF accessible on mobile application*, including downloadable version*
	Offline		* Develop offline contents (e.g. pamphlets) for HCPs and PW
	Overall accessibility		* *Make dose recommendations available in local languages* * Use videos or infographics to make information accessible to patients, especially those with lower literacy
**Sustainability**			*Gain support from end-users and stakeholders to advocate to national funders

*HCP* Healthcare practitioner, *MID* Model-informed dose, *MIPF* Model-informed pregnancy formulary, *PW* Pregnant women, *TIS* Teratology Information Service, *WHO* World Health Organization. The most frequently mentioned barriers and facilitators are outlined in bold. Highlighted in italics are barriers and facilitators specifically mentioned by participants outside the Netherlands or the UK

##### Awareness of pharmacokinetics in pregnancy and relevance of a MIPF

HCPs’ level of awareness of altered pharmacokinetics in pregnancy varied, in part depending on their specialty. Few had questioned the status quo of antenatal dosing before taking part. However, HCPs largely concurred on the potential for pregnancy-adjusted doses to improve the quality of maternal and foetal pharmacotherapy, at least for certain medications.

There’s always just the assumption that the dose is the same as any other adult dose. (clinical pharmacist, INT8 for interview 8, UK)

I find it very promising that you could use a model instead of clinical data to give well-substantiated advice. (general practitioner 2, FG3 for focus group 3, Netherlands)

Primarily noted as a deficiency, was the lack of actionable information on foetal safety, described as a prerequisite for dose selection by several HCPs. Amidst restricted evidence, HCPs’ concerns about foetal harm led many to be conservative when prescribing or dosing antenatal medication:

In practice, most [HCPs] have this sense of ‘do no harm’, meaning that you are more focused on whether the drug is safe. (clinical pharmacist 3, FG6, Netherlands)

##### Credibility, feasibility and acceptability of a MIPF

Although few HCPs were familiar with pharmacokinetic models, MID was mostly perceived as a promising, if ambitious, approach.

It's promising, but it’s such a huge body of work and there are so many variables. (obstetric physician 3, INT3, UK)

Most HCPs indicated that they would be willing to follow model-informed doses as the best available guidance for antenatal medication dosing.

I would follow model-informed doses because there’s probably no better information out there. (anaesthetist 1, FG1, Netherlands)

One requirement for most HCPs however, was that model predictions be verified against clinical data.

I’m happy to work with model-informed doses but I would need evidence points that validate the model. (gynaecologist-obstetrician 9, INT8, the UK)

Given HCPs’ limited ability to interpret modelling evidence, institutional endorsement and a rigorous dose review by multidisciplinary experts, including modellers and clinical end-users, could help build trust in a MIPF according to participants. Priority should be given to widely used medications in pregnancy, preferably across various settings and medical specialties.

It would be helpful if the uncertainty [of a model] can be quantified, both for the doctor and patient. (psychiatrist 2, FG4, Netherlands)

Knowing that respected groups and people have had input and reviewed [the doses] is really reassuring (research physician, INT6, South Africa)

Address diseases that cause the highest maternal mortality and morbidity. (gynaecologist- obstetrician 11, INT11, Kenya)

Participants recommended transparently sharing model assumptions and performance, preferably online, for HCPs seeking more information. HCPs were concerned about pharmacokinetic models providing insufficient information on medication safety and failing to account for individual patient characteristics and comorbidities.

I think it doesn’t go all the way to answer one of the main questions that most women have about the long-term [fetal] outcomes. (midwife 3, INT13, UK)

Such models could however provide useful insights like the impact of physiological variations within different populations on dosing needs.

I see a lot of potential for it globally. It may also start to show differences in how medications are processed by different populations. (gynaecologist- obstetrician 11, INT11, Kenya)

##### Attitude towards dosing considerations and shared decision-making

HCPs expressed different views on dosing considerations and shared decision-making. While pregnant women were deemed likely to demand information on foetal safety, described by many HCPs as their primary concern, a smaller share of pregnant women may be inclined to participate in dosing decisions.

I don’t think that doses are something many patients think about much. (neurologist, INT1, Netherlands)

However, participants felt that pregnant women could gain useful insights from a MIPF if intelligible information was shared with them.

You might want to get information on the fetal exposure to a medication so you can share this information with the mother. (community pharmacist 1, FG5, Netherlands)

With explanations about the model, you’ll get people on your side and enhance treatment adherence. (midwife 3, FG7, Netherlands)

When pregnant women are empowered to know that we have tried to use these models, simplifying what a model looks like, I think they’d be very interested in this information. (clinical researcher, FG8, Uganda)

#### Pregnant women

**Fig. 2:**
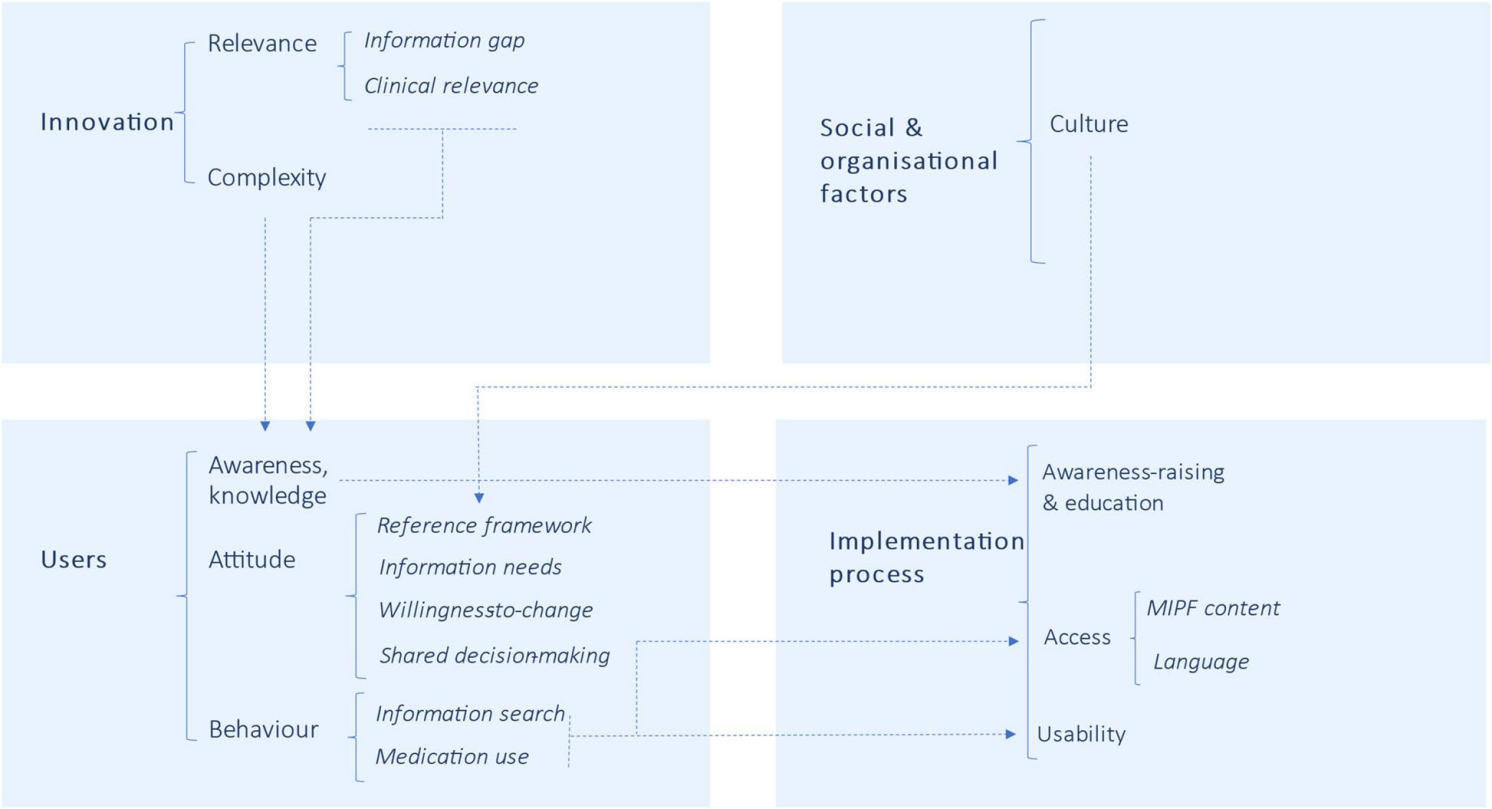
Perceived barriers and facilitators for the implementation of model-informed dosing in pregnancy according to pregnant women. The dotted arrows depict some of the relationships between domains, categories and subcategories of themes

Pregnant women described barriers and facilitators falling under nine categories across the same four domains as HCPs (Table 3 and Fig. 2). Ten categories from Sluisveld et al.’s framework were removed, five were adapted or merged, and one was added (Additional file 4). Additional quotes are listed in Additional file 6.

**Table 3:** Perceived barriers and facilitators for the implementation of model-informed dosing in pregnancy according to pregnant women

Category	Subcategory	Barrier	Facilitator
**Innovation**			
**Relevance**	Information gap	* Missing, contradictory or vague information on medication safety for unborn children is the most important gap * Information on medications during breastfeeding is needed	* MID would help reduce stress from uncertainties around medication use, and sometimes doses * A MIPF would offer information on dosing that some PW are currently missing
	Clinical relevance	* Models do not provide information on the foetal safety of medications	* Models provide information on why different doses may be needed during pregnancy * Models give information on how much medication goes to the baby * Models reduce the need for PW and babies to face the risks of participating in medication research
**Complexity**		* Computer models are hard to understand	
**Users and stakeholders**			
**Awareness, Knowledge**		* Few PW know that evidence for dosing in pregnancy is lacking * Few PW are aware that changes in their body during pregnancy may warrant altered doses	* Information on MIDs can more readily be understood by PW with long-term conditions as they tend to know more on dosing (considerations)
**Attitude**	Reference framework	* **Foetal safety is the primary concern for PW and for HCPs**	* Maternal health is also important according to some PW * By helping to adequately treat maternal disease, MID could significantly improve a PW’s quality of life while better protecting babies from indirect harm * Positive perception of computer models and their applications
	Information needs	* **Reliable information on the foetal safety of medications is needed**	A MIPF would help address certain PW’s desire to have information on: * The considerations behind pregnancy-adjusted doses * MID * The placental transfer of medications
	Willingness-to-change	* Questions about whether higher doses are always needed to reach an effect	* Several PW would be willing to take a higher dose if their HCP recommends it, especially if the reasons have been explained
	Shared decision-making	* PW have mixed opinions on whether they should be involved in making decisions on appropriate doses	* Information on MIDs would help PW who desire to be involved in dosing decisions * PW see themselves as responsible for the (foetal) consequences of medication use, with some desiring greater autonomy in making decisions on medication, including dosing)
**Behaviour**	Information search	* PW consult various sources of information on medication in pregnancy, including online and their HCP	
	Medication use	* **PW prefer avoiding medications if they can to prevent fetal harm**	* PW are more willing to take medication if their symptoms are severe
**Social and organisational factors**			
**Culture**		* Society, including PW's partners prioritise foetal safety	* Decisions on medication in pregnancy have entered public discourse (e.g. Covid vaccine)
**Implementation process**			
**Awareness-raising and education**			* Train HCPs on the need for pregnancy-adjusted doses * PW can demand from their HCPs that they pay attention to the need for adjusted doses during pregnancy * Share information on a MIPF via social media for PW
**Access**		* A website on MID in pregnancy may cause an information overload; some PW would prefer to be informed by their HCP * Few PW know about or have consulted the Dutch TIS website (especially women without a medical background)	* Some PW would consult a website with information on doses in pregnancy
**Usability**	MIPF content		* Several PW would like information on modelling approach * Include information on the placental transfer of medication * **Include information on changes in PW’s bodies and how this may affect dosing needs**
	Language		* Use language that is clear for all PW regardless of the education level

*HCP* Healthcare practitioner, *MID* Model-informed dosing, *MIPF* Model-informed pregnancy formulary, *PW* Pregnant women, *TIS* Teratology Information Service. The most frequently mentioned barriers and facilitators are outlined in bold. Highlighted in italics are barriers and facilitators specifically outlined by women with a medical background

##### Information gap

All pregnant women regardless of their medication use had sought information on medication during pregnancy. Their searches primarily targeted medication use, and in some cases medication dosing. Information was searched online or by consulting their HCP. The obtained information was often described as discrepant, vague or unclear.

I feel that there’s still little certainty about both [appropriate] types of medication and medication doses. (pregnant woman 3, with anxiety disorder, FG11)

Everything that I have read is very, very superficial and feels like it doesn’t help me at all. (pregnant woman 11, with epilepsy, INT15)

While most women described the lack of information on foetal safety as a crucial knowledge gap that may create unwanted stress, a few indicated an interest in dosing and the maternal efficacy of medications, if only as a means to protect their baby from indirect harm.

It’s very important to know the extent to which the medication is going to affect the baby and how much it affects the mother too. (pregnant woman 10, INT14)

##### Clinical relevance of model-informed dosing and willingness-to-follow model-informed doses

MID, while complex and new, was perceived as a much needed and promising approach to improve care for pregnant women and their unborn children, for example by providing information on the placental transfer of medications and by limiting the need for pregnant women and their foetuses to undergo pharmacological research.

There’s so much that is done by models these days that it’s funny that this isn’t the case yet in the medical world. (pregnant woman 1, FG11)

This is an elegant way to collect all the data, meaning, not as part of a clinical study but from behind a desk. (pregnant woman 5, FG11)

A majority of women appeared willing to follow model-informed doses in pregnancy.

##### Awareness and attitude towards dosing and shared decision-making

Respondents were mostly unaware of the lack of specific evidence for dosing in pregnancy. Some pregnant women indicated that they wanted to be informed — either by their HCP and/or online — and involved in decisions in this regard, others preferring to leave dose selection to HCPs.

Dosing is for the doctors. (pregnant woman 10, INT13)

I want to know when my dose must be increased. (pregnant woman 6, INT14)

One woman identified HCPs’ disproportionate focus on medication safety as a barrier for MIPF adoption.

I think that doctors should first realise how much [a poorly treated condition] may impact the pregnancy and a woman’s happiness. Otherwise, they won’t invest time in understanding this [approach] better. (pregnant woman 11, with epilepsy, INT15)

## Discussion

### Main findings

This qualitative study revealed multiple perceived barriers and facilitators for the implementation of model-informed dosing in pregnancy. Most participants in both groups perceived the formulation of specific, evidence-based doses in pregnancy as a valuable addition to antenatal pharmacotherapy, described by many as suboptimal. While a majority of healthcare practitioners and pregnant women appeared willing to follow antenatal model-informed doses, they also identified several barriers that need addressing to support the adoption of a model-informed pregnancy formulary in clinical care. A key constraint according to both groups of stakeholders was the limited ability of models to provide information on the foetal safety of medications. According to HCPs, important considerations to scale the proposed approach included model validation, transparency on model uncertainties and endorsement of the recommended doses by trusted organisations and multidisciplinary experts. While HCPs’ views on the need for patient information differed, several pregnant women indicated that they wanted to be informed, or even partake in shared decision-making on antenatal doses.

### Interpretation

Despite the insights gained from the growing use of maternofoetal pharmacokinetic models [5, 8], so far, model-informed dosing has not been implemented in antenatal care. The few available expert reviews on the clinical implementation of MID, none of which covered pregnant women as a target population, mostly identified technical barriers and enablers for MID, some of which aligned with factors outlined in our study. Examples include the need for transparent model validation and the limited availability of alternative medication formulations [9, 10, 18, 19]. The potential of a multi-stakeholder approach to implementation, involving end-users, was also identified previously [11]. Given the focus of prior studies on precision dosing rather than the development of dosing guidelines for a patient population, as intended by the MIPF, many barriers and facilitators identified in this study were new.

The present study, furthermore, sheds new light on several themes of importance with regards to medication dosing in pregnancy, an area of limited research [4]. First was the uncertainty characterising pharmacological decision-making in pregnancy, given often unclear and potentially misaligned risks and benefits of medications for mother and child. Traditionally centred on medication use [20, 21], discussions about the risk–benefit balance for determining suitable antenatal doses were perceived as arduous by HCP participants. Among other factors, this complexity arose from most HCPs’ limited knowledge of pharmacokinetics and the poor availability and accessibility of supportive evidence. Conveying such uncertainties to pregnant women was considered an even greater challenge by HCPs. Second was the greater weight attributed to foetal safety compared to maternal well-being by many HCPs and some pregnant women irrespective of the setting. Both groups, as well as several studies [22–24], have described how concerns over potential foetal risks sometimes prevailed over maternal efficacy and led to conservative prescribing and dosing practices by clinicians. HCPs’ concerns over liability, prevalent in obstetric care [25, 26], contributed to this further. These various elements were deemed to feed into suboptimal pharmacological care in pregnancy according to multiple HCPs and pregnant women in this study. Several of them were hopeful that the use of pharmacokinetic models, as an additional source of evidence alongside clinical studies, would bring improvements in this regard, alongside a broader shift in culture around antenatal medication prescription and dosing.

### Strengths and limitations

To our knowledge, this is the first study to explore the perceived barriers and facilitators for implementing MID in pregnancy according to healthcare practitioners and pregnant women. A diverse group of HCPs from various countries and settings, and pregnant women from the Netherlands, were consulted to assess the perceived feasibility and acceptability of MID in pregnancy. HCPs from numerous specialties involved in making decisions or counselling pregnant women on medication dosing were represented.

HCP participants to this study had varied levels of familiarity with clinical pharmacology and pharmacometrics. Although our sampling strategy combined recruitment of experts in these fields with broader channels targeting all medical specialists caring for pregnant women, it may have yielded participants who were more acquainted with MID than average physicians. However, most HCP interviewees indicated no prior knowledge of pharmacokinetic models. While a geographically diverse set of HCP participants was sought, most interviewees were recruited in the Netherlands. Participation from HCPs in the UK and several African and Asian countries provided additional insights into how the perceived barriers and enablers for a MIPF may vary internationally and across socio-economic and sociocultural settings. Although incorporating these varied perspectives enhanced our understanding of factors that may influence the adoption of model-informed antenatal doses beyond the Netherlands, this qualitative study did not aim to achieve an internationally representative sample of HCPs [27]. Overall, while recurring themes were identified by participants regardless of location, a small number of barriers and facilitators were specifically mentioned by HCPs working in lower-and-middle-income countries. Barriers included limited availability of certain medications and medication formulations, and the varied levels of medical training of HCPs, with healthcare workers in more remote settings often receiving more basic training. In addition, participants from lower-and-middle-income countries shared distinct preferences regarding appropriate dissemination channels for model-informed antenatal doses, highlighting the need to develop offline contents, including visual materials, for patients with low literacy levels, and to make dose recommendations available in various local languages.

Participation from pregnant women was restricted to the Netherlands due to ethics requirements, and five of eleven participants in this group had a medical background, with higher-than-average educational levels overall. Given self-selection and a high degree of health literacy, participating pregnant women may possess greater knowledge and interest in medication dosing compared to the average pregnant woman. The information needs of pregnant women in our sample did not appear to differ based on whether they possessed a medical background. Most women highlighted the vague and sometimes contradictory nature of available information on medication in pregnancy. The awareness of rationales for altered dosing and the extent of information desired varied among pregnant women, irrespective of their medical background. The presence of a chronic condition seemed to be of greater influence in this regard; all pregnant women with such conditions indicated that they wanted to be informed about the dose received and its underlying evidence.

Overall, considering the characteristics of HCPs and pregnant women recruited for this study, selection bias, potentially manifesting as a higher or lower willingness to rely on MID and differentiated information needs, may have been present. For the most part, it could be observed that more knowledgeable participants tended to be more critical towards the use of MID in pregnancy and had more stringent information requirements on model-informed doses and underlying evidence.

### Implications and lessons learned

Insights from this study will guide the development of the envisioned MIPF as part of project MADAM following a participatory design approach [28]. Key design objectives, in line with study findings, include obtaining endorsement of the proposed MID methods from professional societies and ensuring transparent communication on dosing rationales, including on model assumptions and quality, within the broader context of available evidence. This information should be accessible to both HCPs and pregnant women at an appropriate level of comprehension. Furthermore, raising understanding about reasons for pregnancy-adjusted doses among HCPs and pregnant women, many of whom had little awareness of this matter, could be a critical intervention to modify antenatal dosing practices. The potential benefits and limitations of MID in addressing the perceived lack of information on the foetal safety of medications should be clearly outlined. Applicability of the obtained results to a wider range of HCPs and pregnant women will be investigated through an international survey among both these groups, conducted as a second step of this qualitative study. Additional perspectives from other international stakeholders such as pregnant women's partners, medical ethicists, regulators and health policy makers could be gained to support the implementation of model-informed antenatal doses in various settings. 

## Conclusions

Despite the perceived novelty of model-informed dosing in pregnancy, this qualitative study revealed a high willingness to embrace this approach among healthcare practitioners and pregnant women. This was particularly true given the observed scarcity of evidence to support maternal and foetal pharmacotherapy, which some participants perceived as suboptimal. Key prerequisites for implementing model-informed doses in pregnancy included quality insurance through institutional endorsement, transparency on model validation and patient information. Given the large proportion of participants from the Netherlands and of pregnant women with a medical background, perceived barriers and facilitators for model-informed dosing in pregnancy may be further explored among healthcare practitioners and pregnant women more widely. Participation of pregnant women with varied educational backgrounds should be prioritised. This qualitative research will be used to inform the design of a model-informed pregnancy formulary for use in routine antenatal care.

### Supplementary Information

Additional file 1. Topic guides.

Additional file 2. Standards for Reporting Qualitative Research Checklist.

Additional file 3. List of focus groups and interviews and participant characteristics.

Additional file 4. Adapted frameworks from hybrid inductive and deductive analysis.

Additional file 5. Quotes from healthcare practitioners.

Additional file 6. Quotes from pregnant women.

## Data Availability

Detailed quotes from the focus groups and interviews and other supporting data are available as supplementary information. Anonymised transcripts of the focus groups and interviews are available from the corresponding author on reasonable request.

## Abbreviations

HCP: Healthcare practitioner
MIPF: Model-informed pregnancy formulary
