# Supplementary material for: Perceived barriers and facilitators for model-informed dosing in pregnancy: a qualitative study across healthcare practitioners and pregnant women
Source: BMC Med. 2024 Jun 18;22:248. doi: 10.1186/s12916-024-03450-8 (PMC11184760; doi:10.1186/s12916-024-03450-8)
Supplement: Supplementary file 1 — Additional file 1. Topic guides. [file 12916_2024_3450_MOESM1_ESM.docx]

**Additional file 1 – Topic guides**

- 1. **Healthcare practitioners (Netherlands)**

* The latter text, originally in Dutch, was translated on 15/9/2023 using the DeepL translator (<https://www.deepl.com/translator>).

Introduction

- Welcome

o Thank you for joining this conversation

o Introduction of moderators

o Together with MUMC+, Lareb and the Gates Foundation, looking at medication dosages in pregnancy. Our group is looking at the role of computer models combined with placental perfusion data as new evidence for dosage recommendations in pregnant women. The long-term goal is to establish an evidence-based pregnancy formulary (PF)

o As a physician I am responsible for the translation of model results to the clinic. It is important for us that the PF meets the needs of users = reason I speak to you, also to anticipate barriers in implementing a PF partly based on computer models

- Goal today

o Hear your perspective as health care providers on 1) the existing evidence & information for medication dosing in pregnancy and 2) your views on using computer models as new evidence.

o I will do this using some questions that you can discuss together

- Agenda

o Conversation takes 1 hour and 15 minutes > finished around 21.15 ; I am here as moderator

The session has 3 parts, namely

1 Questions regarding the existing information on medication dosages in pregnancy

2 Presentation on the use of computer models as new evidence for this

3 Questions regarding this new type of evidence

- A few conversation rules

(1) I will record the conversation including video footage for analysis; should you be more comfortable, turn your camera off; video footage will be deleted after transcription; transcripts anonymized

(2) For the first question I ask you all to give an answer; after that you may think for yourself whether you want to answer a question or not; participation is very much appreciated; this is not about knowledge so there are no wrong answers

(3) Takwyour turn = you don't have to wait for me to give you a turn. Respmdomg to each other's answers is encouraged so that a discussion can arise

(4) I may sometimes interrupt you to introduce a new question or to ask further questions

(5) You are here as a healthcare provider but if you want to share personal information and experiences you may do so; information discussed here is confidential

(6) Logistics You don't have to mute in between sessions but do so if there is noise in the background

(7) You may but you certainly do not have to share personal or medical information with the group

(8) I.mportant that what is discussed here is confidential; the information is stored in such a way that it cannot be traced back to anyone

o Questions?

Questions prior to the presentation

1- Can you introduce yourself (areas of interest, experience with medication dosages in pregnancy) and tell why you decided to join this group discussion? Everyone 1 turn

2- What do you think about the current information on medication dosages in pregnancy in terms of supporting your work in practice?

- What sources do you use? What do you think of the completeness/reliability/usability of these sources?

- What are your thoughts of the evidence for medication dosages during pregnancy?

3- Do you encounter any challenges when trying to recommend a particular medication dosage to 1 pregnant person?= Current way of dealing with medication dosages in pregnancy

- What would you need (in an ideal world)

4- Are you familiar with the use of computer models as evidence for dosing recommendations (in general / for specific patient groups)?

PRESENTATION

6.To what extent would you be confident / willing to use dosing recommendations drawing on computer models? What are conditions (= how much information do you need about the model) for you to follow such doses ?

7. What are your views on the uses for this evidence? What kind of information would you like to get from these models (to support your daily practice), if any?

- For yourself

- For patients

8. Do you anticipate any barriers in implementing a pregnancy formulary based on computer models?

9. What might help us in implementing a pregnancy formulary?

10. To what extent do you discuss the evidence behind medication choices and/or dosages with prospective parents and involve them in this decision? Would you then also discuss models with them?

11. What are certain medication groups that you would like to see included in a pregnancy formulary

Conclusion

- This brings us to the end of the conversatin

- Thank you all for your participation; heard valuable information (summarise some themes)

- Next steps for us:

o Several more focus groups to be conudcted with different specialties and pregnant women

o This conversation will be analysed; goal: identify key themes before using the latter themes to design an international online survey

o We hope to publish this data

- If you would like to contribute/share other ideas about this, please contact us, will share email.

- Any questions or comments following this talk**?**

**1.2. Healthcare practitioners (UK, low- and middle income countries)**

This is a concise topic guide. The introduction and conclusion are similar to version 1.1.

1- Could you introduce yourself (specialty, experience with medication dosages during pregnancy) and explain why you decided to participate in this group conversation?

2- What are your opinions on the current information about medication dosages during pregnancy? What challenges do you encounter when prescribing a specific medication dosage for a pregnant woman?

- Sources of information
- Quality of the sources?
- Satisfaction with the sources as a user?

3- Who would be pregnant women’s first point of care for medication in pregnancy in Kenya?

4- Shared decision-making: Do you discuss medication with pregnant women, and if so, to what extent do you discuss medication doses in pregnancy?

5- How much knowledge do you have on how dose recommendations during pregnancy are established?

6- Are you familiar with the use of computer models as evidence for dosage recommendations (in general / for specific groups)?

PRESENTATION

7 What are your first impressions on model-informed dosing? Feel free to ask questions and to be critical.

8 Do you see an added value in disseminating the use of MID in pregnancy, and if you do, how so and for whom?

9 Would you trust model-informed doses and are there any key requirements for you to follow such recommendations?

10 What are your thoughts on the involvement of an editorial board? Do you have any suggestions as to the profiles of experts that we have recruited?

11 What information would you need to be willing to follow a model-informed dose? In terms of:

- Dose

- Evidence including on pharmacokinetic models

12 What would be your preferred source/format for accessing the MIPF?

13 Who should have access to this information?

14 Do you expect any challenges in setting up and disseminating the use of a model-informed pregnancy formulary (MIPF) ?

15 What could help disseminate the MIPF among users and stakeholders?

16 Which medications would you include first and why?

- 1. **Pregnant women (Netherlands)***

* The latter text, originally in Dutch, was translated on 15/9/2023 using the DeepL translator (<https://www.deepl.com/translator>).

Introduction

- Welcome

o Thank you for joining conversation

o Introductions moderator:

Together with MUMC+ and Lareb Moeders van Morgen, o into medication dosage during pregnancy; specifically whether computer models can be used as a new form of evidence to make special dosage recommendations in pregnant women.

o Idea of project (MADAM) = create a new resource so that healthcare providers (physicians and pharmacists) to better advise pregnant women on medication dosages during pregnancy

o Important for us that this resource also meets the needs of health care providers but also pregnant women it is about; this is the reason I am speaking to you

- Goals

o You are here because you are either pregnant or have recently been pregnant & wanted to participate

o Goal of today: discuss your perspectives on a number of questions around a) using medication during pregnancy, b) providing information about it c) decision making with your doctor d) new form of evidence that I will tell you about

o The conversation will be recorded and transcribed so we can identify important themes; the footage will be deleted after transcribing; if you are uncomfortable, your camera may be turned off

Agenda

o FG lasts 1 hour 15 min > finished around 21.15

o I am here as a moderator

o The session has 3 parts, namely

- Questions about the current situation regarding medication in pregnancy
- Short presentation on the use of computer models as evidence for doses in pregnancy
- Your perspectives in this regard

- A few rules for the discussion (if something is not clear > interrupt me)

(1) There is no right or wrong answer. No prior knowledge needed either. Nice to hear your real opinion and different opinions are welcome.

(2) You may always decide not to answer a question; I will ask everyone to participate for the 1st question (≈ introduction round); hereafter participation is appreciated but not required

(3) Take your turn; feel free respond to each other's so that a discussion can emerge

(4) Because I do want to discuss a number of different questions with you, I may sometimes interrupt you to introduce a new question

(5) You may but you certainly do not need to share personal or medical information with the group;

(6) Very important: what is discussed here is confidential = should not be shared with others; we store information in such a way that it cannot be traced to anyone

(7) You don't have to mute your microphones but please do so if there is background nose

- Questions?

1 Introductions: name and what made you decide to join this conversation = why are medications in pregnancy an important topic for you?

2 Would anyone like to share something about an experience of their own?

3 There are different sources of information about medicines in pregnancy. Which sources do you use and how useful and user-friendly did you find these sources?

- Doctors, other healthcare providers

- Internet (what websites? apps?)

- Books, magazines

- Family, friends

- Social media

- What is your preference regarding the source of information?

4 What kind of information do you look up when it comes to medication during pregnancy?

- Benefits l risks l safety

- Evidence?

- Medication dosage?

Do you have any unanswered questions on these various aspects?

5 What are important considerations when making decisions regarding medication during pregnancy?

E.g. benefits/risks of medications during pregnancy? For whom?

E.g. medication dosages?

6 What makes it feel safe to take a medication during pregnancy? How confident do you feel when you have to make decisions ?

7 Who should make a decision about using medication during pregnancy, who is responsible? To what extent do you want to be involved (discuss the provision of information and making decisions)? What about medication dosages?

PRESENTATION

8 What are your initial thoughts after this presentation? Any questions, uncertainties?

9 How do you view using computer modelling as new evidence for medication dosing during pregnancy?

- Do you see advantages?

- Do you see disadvantages?

10 Suppose these models become a new form of evidence for medication dosing in pregnancy and you are considering using a medication yourself, to what extent would you like to have information about models? If you feel the need to get information, what kind of information?

11 Through which channels would you prefer to obtain this information? What are your thoughts about a website with or without a section for parents? Would you consult such a website

Conclusion: - This brings us to the end of the conversatin

- Thank you all for your participation; heard valuable information (summarise some themes)

- Next steps for us:

o Several more focus groups to be conudcted with different specialties and pregnant women

o This conversation will be analysed; goal: identify key themes before using the latter themes to design an international online survey

o We hope to publish this data

- If you would like to contribute/share other ideas about this, please contact us, will share email.

- Any questions or comments following this talk**?**
