# Supplementary material for: Perceived barriers and facilitators for model-informed dosing in pregnancy: a qualitative study across healthcare practitioners and pregnant women
Source: BMC Med. 2024 Jun 18;22:248. doi: 10.1186/s12916-024-03450-8 (PMC11184760; doi:10.1186/s12916-024-03450-8)
Supplement: Supplementary file 3 — Additional file 3. List of focus groups and interviews and participant characteristics. [file 12916_2024_3450_MOESM3_ESM.docx]

**Additional file 3 – List of focus groups and interviews and participant characteristics**

AH: academic hospital, E: East Netherlands, FG: focus group, GH: community hospital, M: man, Mi: middle region

INT: interview, NL: Netherlands, NW: North-West Netherlands, OC: outpatient care, SA: South Africa, SW: South West Netherlands, UK: United Kingdom, W: woman * Only indicated for healthcare practitioners in the Netherlands. ** Only indicated for HCPs. *** Clinical pharmacologist

| **Focus group or interview code** | **Date** | **Country of work or residence of participants (language of interview)** | **Profession and other characteristics of participants (gender, region,* type of setting)** | **Participant code** |
| --- | --- | --- | --- | --- |
| **HCPs** | | | | |
| **FG1** | 5/1/22 | NL (NL) | Obstetric anaesthetist (W, E, GH)  Obstetric anaesthetist, (W, SE, GH)  Psychiatrist with obstetric expertise  (W, Mi, GH) | Anaesthetist 1  Anaesthetist 2  Psychiatrist 1 |
| **FG2** | 6/1/22 | NL (NL) | Clinical pharmacist (W, Mi, GH)  Clinical pharmacist (M, SE, GH)  Clinical pharmacist (M, N, GH)  Clinical pharmacist in training (M, N, AH) | Clinical pharmacist 1  Clinical pharmacist 2  Clinical pharmacist 3  Clinical pharmacist 4 |
| **FG3** | 10/1/22 | NL (NL) | General practitioner (W, E, OC)  General practitioner (M, E, OC)  General practitioner (M, M, OC)  General practitioner (W, M, OC)  General practitioner in training (W, S, OC) | General practitioner 1  General practitioner 2  General practitioner 3  General practitioner 4  General practitioner 5 |
| **FG4** | 11/1/22 | NL (NL) | Internist in training (M, SW, AH)  Oncologist (W, SW, AH)  Psychiatrist (M, E, AH) | Internist 1  Internist 2  Psychiatrist 2 |
| **INT1** | 13/1/22 | NL (NL) | Neurologist (W, NE, GH) | Neurologist |
| **INT2** | 14/1/22 | NL (NL) | Anaesthetist 3 (W, SW, AH) | Anaesthetist 3 |
| **FG5** | 18/1/22 | NL (NL) | Community pharmacist (W, E, OC)  Community pharmacist (W, E, OC)  Community pharmacist (W, E, OC) | Community pharmacist 1  Community pharmacist 2  Community pharmacist 3 |
| **FG6** | 20/1/22 | NL (NL) | Gynaecologist-obstetrician (W, NW, AH)  Gynaecologist-obstetrician (M, SW, AH)*  Gynaecologist-obstetrician (M, NW, AH)  Gynaecologist-obstetrician (W, SW, AH)  Gynaecologist-obstetrician (W, E, AH)  Gynaecologist (W, NW, GH)** | Gynaecologist 1  Gynaecologist 2  Gynaecologist 3  Gynaecologist 4  Gynaecologist 5  Gynaecologist 6 |
| **INT3** | 20/1/22 | NL (NL) | Gastro-enterologist (W, N, AH) | Gastro-enterologist |
| **FG7** | 10/2/22 | NL (NL) | Midwife (W, SE, AH)  Midwife (W, SE, AH)  Midwife (W, E, OC) | Midwife 1  Midwife 2  Midwife 3 |
| **INT4** | 27/2/22 | NL (NL) | Clinical pharmacist (M, SW, AH) | Clinical pharmacist 5 |
| **INT5** | 21/10/22 | NL (NL) | Clinical researcher & implementation expert (W, SW/E, AH) | Clinical researcher 1 |
| **INT6** | 21/12/22 | Vietnam, India, UK (EN) | Gynaecologist-obstetrician (W, AH & GH) | Gynaecologist 7 |
| **FG8 and FG10** | 21/12/23  17/1/23 | Uganda (EN) | Internist** (W, AH)  Research pharmac(olog)ist (W, AH)  Clinical pharmacist (W, AH)  Clinical researcher (W, OC) | Internist 3  Research pharmac(olog)ist  Clinical pharmacist 6  Clinical researcher 2 |
| **INT7** | 22/12/23 | UK (EN) | Clinical pharmacist (W, AH) | Clinical pharmacist |
| **FG9** | 11/1/23 | SA (EN) | Foundation doctor (W, GH)  Foundation doctor (W, GH)  Gynaecologist-obstetrician in training (W, AH) | Junior doctor 1  Junior doctor 2  Gynaecologist 8 |
| **INT8** | 12/1/23 | UK (EN) | Gynaecologist-obstetrician (W, AH) | Gynaecologist 9 |
| **INT9** | 23/1/23 | UK (EN) | Obstetric physician (W, AH) | Obstetric physician 1 |
| **INT16** | 25/1/23 | SA (EN) | Clinical pharmacologist (W, AH)  Research physician (W, AH) | Clinical pharmacologist  Research physician |
| **INT10** | 3/2/23 | Cameroon (EN) | Gynaecologist-obstetrician (M, AH) | Gynaecologist 10 |
| **INT11** | 27/2/23 | Kenya (EN) | Gynaecologist-obstetrician (W, GH) | Gynaecologist 11 |
| **INT12** | 8/3/23 | UK (EN) | Obstetric physician (W, AH) | Obstetric physician 2 |
| **INT13** | 27/3/23 | UK (EN) | Obstetric physician (W, GH)  Midwife (W, AH) | Obstetric physician 3  Midwife 4 |
| **Pregnant women** | | | | |
| **FG11** | 2/2/22 | NL (NL) | Pregnant woman (E)  Pregnant woman (Mi)  Pregnant woman (Mi, nurse, anxiety disorder)  Pregnant woman (SW)  Pregnant woman (E, bipolar disorder & asthma) | Pregnant woman 1  Pregnant woman 2  Pregnant woman 3  Pregnant woman 4  Pregnant woman 5 |
| **INT14** | 10/2/22 | NL (EN) | Pregnant woman (Mi, Iranian) | Pregnant woman 6 |
| **FG12** | 17/2/22 | NL (NL) | Pregnant woman (NW, paediatrician in training)  Pregnant woman (SW, gynaecologist)  Pregnant woman (SW, gynaecologist in training)  Pregnant woman (Mi, psychiatrist) | Pregnant woman 7 / paediatrician in training  Pregnant woman 8 / gynaecologist 12  Pregnant woman 9, gynaecologist in training  Pregnant woman 10 / psychiatrist 3 |
| **INT15** | 31/3/22 | NL (NL) | Pregnant woman (SW, epilepsy) | Pregnant woman 11 |
