# Supplementary material for: Perceived barriers and facilitators for model-informed dosing in pregnancy: a qualitative study across healthcare practitioners and pregnant women
Source: BMC Med. 2024 Jun 18;22:248. doi: 10.1186/s12916-024-03450-8 (PMC11184760; doi:10.1186/s12916-024-03450-8)
Supplement: Supplementary file 4 — Additional file 4. Adapted frameworks from hybrid inductive and deductive analysis. [file 12916_2024_3450_MOESM4_ESM.docx]

**Additional file 4 – Adapted frameworks from hybrid inductive and deductive analysis**

Domains are indicated in bold. Categories are described in regular text. Categories in italics in van Sluisveld’s *et al*.’s framework did not appear in the final framework. Underlined categories in the final framework were new.

| Framework Van Sluisveld’s et al. | Framework from hybrid inductive and deductive analysis | |
| --- | --- | --- |
|  | Healthcare practitioners | Pregnant women |
| Intervention   - Advantages in Practice - Usefulness - Accessibility - Credibility - Feasibility - *Attractiveness* - *Contradictory Guidelines* | **Innovation**   - Knowledge gap - Clinical relevance - Complexity - Credibility - Feasibility | **Innovation**   - Relevance - Complexity |
| Professional   - *Cognition* - Awareness* - Knowledge* - Attitude - Behavioural Routines - *Motivation* - *Skills* | **Users and stakeholders**   - Awareness, knowledge - Attitude - Behaviour | **Users and stakeholders**   - Awareness, knowledge - Attitude - Behaviour |
| Patient   - Compliance - Knowledge - Skills - Attitude - Preferences |  |  |
| Social   - Collaboration* - Leadership* - Culture of social network - *Opinion of colleagues* | **Social & organizational factors**   - Organization - Culture - Legal context | **Social & organizational factors**   - Culture |
| Organisational   - Organization of care processes* - Organizational structure* - Time* - Staff* - Capacities* - Resources* - Structure* - Technical Support* |  |  |
| Society   - *Social developments* - *Political developments & policies* - Legal obligations & regulations - Financial arrangement - Moral objections* |  |  |
| Implementation   - Clarity* - Support* - Accessibility | **Implementation**   - Awareness-raising & education - Acceptability & usability - Access - Sustainability | **Implementation**   - Awareness-raising & education - Usability - Access |

Reference: van Sluisveld et al., 2017. A strategy to enhance the safety and efficiency of handovers of ICU patients: study protocol of the pICUp s tudy. Implementation Science 2013 8:67.
