# Supplementary material for: Perceived barriers and facilitators for model-informed dosing in pregnancy: a qualitative study across healthcare practitioners and pregnant women
Source: BMC Med. 2024 Jun 18;22:248. doi: 10.1186/s12916-024-03450-8 (PMC11184760; doi:10.1186/s12916-024-03450-8)
Supplement: Supplementary file 5 — Additional file 5. Quotes from healthcare practitioners. [file 12916_2024_3450_MOESM5_ESM.docx]

**Additional file 5 – Quotes from healthcare practitioners**

**Quotes from healthcare practitioners on their perceived barriers and facilitators for the implementation of a model-informed pregnancy formulary –** FG: focus group; HCP: healthcare practitioner, INT: interview; MHRA: Medicines and Healthcare Regulation Authority; MID: model-informed dose; MIPF: model-informed pregnancy formulary, PBPK: physiologically-based pharmacokinetic model, SSRI: selective serotonin reuptake inhibitor, TDM: therapeutic drug monitoring, TIS: Teratology Information Service.

Quotes in Dutch were translated in English.

1. INNOVATION

| Relevance | Knowledge gap | Fetal safety and medication use in pregnancy  *‘I am primarily looking for information on whether something is or is not safe so more of a yes-no question than about medication dose*.’ (internist 2, FG4, Netherlands)  *‘What I find most difficult is that people understandably really want to know what the potential consequences* (of using medication in pregnancy*) are, and you have to say, I don’t know.’* (gynaecologist 2, FG6, Netherlands)  *‘The two main sources of information for starting or stopping medication* (in pregnancy) *are anecdotal evidence and clinical experience. That is a large knowledge gap for a large group of patients.’* (internist 1, FG4, Netherlands)  *‘The lack of knowledge on psychotropics use in pregnancy is an important gap that I’m faced with in my daily practice (…). There’s very little knowledge not only in primary care but also among my colleagues on what can and cannot (be used).’* (psychiatrist 2, FG4, Netherlands)  *‘What I mostly struggle with in practice is the lack of consistent recommendations on medication* (use) *in pregnancy, for example when a patient comes with a medication leaflet that doesn’t align with advice from the Dutch TIS.*’ (general practitioner 4, in training, FG3, Netherlands)  *‘I still have patients that go to the pharmacy and are told that a given drug cannot be used in pregnancy. (…) There are many contradicting stories which creates a lot of unrest.’* (gastroenterologist, INT3, Netherlands)  *‘Women in pregnancy. We know they're underserved (…) with regards to medication in pregnancy.’* (obstetric physician 3, INT13, UK)  ‘*We fill in a lot of queries (from pregnant women). And I’d say medication is probably one of the most frequent questions that we're (…) getting*. *There's such a lack of any concrete information, even you know the more recent resources we have like UKTIS and Bumps (…), it's still gives a very, very woolly sort of explanation to women as to their options’* (midwife 4, INT13, UK)  *‘Whenever I have conversations with specialist colleagues, (…) they will bulk with horror that I’ll be advocating for a certain medication based upon the minimal exposure in pregnancy because they're used to a world where they're operating with trials that have had hundreds if not thousands of people exposed to a certain medication, not just to know its safety profile but also its efficacy.’* (obstetric physician 3, INT13, UK)  Medication doses in pregnancy  *‘There are no doses in guidelines. This is a gap.’* (gynaecologist 4, FG6, Netherlands)  *‘As a pharmacist I have the feeling that I don’t have enough information (…) to make informed decisions on medication dose adjustments* (in pregnancy)*.’* (community pharmacist 1, FG5, Netherlands)  ‘*I feel that dosing in pregnancy is a very poorly developed field of pharmacology, certainly compared to other fields within our specialty.’* (community pharmacist 1, FG5, Netherlands)  *‘X wrote a local guideline about the use of intrathecal morphine (…) for which we (looked at information from) a few countries on doses, concentrations etc. And there was virtually no information on this.’* (anaesthetist 2, FG1, Netherlands)  *‘You cannot expect from a doctor that they decide on their own that a dose must be increased by a third* (in pregnancy).*’* (clinical pharmacist 5, INT4, Netherlands)  *‘There are a few items like HIV drugs. We have established that we must monitor* (the blood concentration) *to see if we can reach sufficiently high levels to maintain a low viral load during pregnancy. (…) Then there’s anti-epileptics and then there’s a big gap.’* (clinical pharmacist 1, FG2, Netherlands)  *‘We know very little about a lot of the* (anti-epileptic) *drugs. We then say that we don’t need to do anything about the dose but that’s a bit shortsighted.’* (neurologist, INT1, Netherlands)    *‘I'm not really aware of many drugs where we know that we need to change the dose, but that's also worrying. (…) I just worry that sometimes the question has not been asked.’* (internist 3,* FG8, Uganda)  *‘What I think is lacking is good evidence. I often struggle (…) with resources or a specific formulary pertaining to pregnant women is not readily available. Generally it's the hassle and bustle of going back and trying to sift through the literature yourself.’* (clinical pharmacologist, INT16, South Africa)  Physiological changes in pregnancy  ‘*What I miss most on the Dutch TIS and the Dutch Formulary is information on physiological changes in pregnancy.’* (internist 1, FG4, Netherlands)  *‘We know that the clearance is very different (*in pregnancy) *but we don’t do anything with this knowledge. It’s weird that we don’t translate this into practice.’* (clinical pharmacist 4, FG2, Netherlands) |
| --- | --- | --- |
|  | Clinical relevance | Evidence-based dosing in pregnancy  *‘I think that everyone (…), is waiting for a reference* (for dosing in pregnancy) *like the Dutch paediatric formulary.‘* **(community pharmacist 1, FG5, Netherlands)**  *‘You just notice that in general there is much need for evidence-based doses. On the other hand, if* (the lack of evidenced-based doses in pregnancy) *caused significant problems, people would have woken up earlier. I am a bit torn between these two thoughts. Apparently it all goes well with antibiotics and those* differences *between (pregnant women and non-pregnant adults)* *don’t matter that much.’* **(clinical** **pharmacist 3, FG2, Netherlands)**  *‘I don't know if the results of all of this will prove that the dosing regimens should be very different. Actually, we might simply prove that actually we're pretty close to where we should be already. But I think that's still helpful information.’* **(obstetric physician 1, INT9, the UK)**  Enhanced care for pregnant women  *‘This a great initiative through which I hope we can reduce the number of medication errors and improve the quality of care for women.’* (**internist 2, FG4, Netherlands**)  ‘*Are there medications where you could provide information upfront that would reduce the burden on pregnant women e.g. by helping meet* (concentration) *targets quicker or by reducing the need for monitoring?’*  **(clinical** **pharmacist 2, FG2, Netherlands)**  ‘(Pregnant) *women may take two paracetamols for a headache but if they hear afterwards that this wasn’t effective at all, then it’s actually a shame to have taken these two paracetamols all those times. I think that an increased dose could make sense in those cases.*’ (**gynaecologist 12, FG12, Netherlands)**    *‘I think that it’d be very helpful if pregnant women were more willing to take medications that are frequently needed and that they’d really like to know that these actually have an effect (…) based on (…) a more targeted dosing strategy.’* **(gynaecologist 12, FG12, Netherlands)**  ‘*I think it sounds excellent. Sounds very needed.*’ **(gynaecologist 7, INT6, Vietnam, India, UK)**  *‘With explanations about the model, you’ll also get people on your side and enhance treatment adherence.’* **(midwife 3, FG7, Netherlands)**  *‘Actually treating the mother properly is better than half treating them.’* (**clinical pharmacist, INT7, UK)**  *‘As a clinician, obviously I'm more interested in the outcomes than the pharmacokinetics of it all, but if that helps to understand a better rationale for dosing, if it helps to optimise dosing so that at least if you're giving drugs to pregnant women, you're doing it in an optimal way to make them healthy then that's a good thing*.’ (**obstetric physician 2, INT12, UK)**  *‘Patients want it, we need to do it.’* **(gynaecologist 9, INT8, UK)**  ‘*If you're empowering the midwives and the people who are gonna be seeing these women first of all, that's really important. It goes a lot further really to have this kind of actual concrete data.’* **(midwife 4, INT13, UK)**  *TDM*  *‘It’s a dilemma. On the one hand, I would like to have as much guidance as possible on medication dosing in pregnancy (…). On the other hand, if the concentration of a drug is so important and it’s possible to measure it, would you not want to measure the concentrations of medications in all pregnant women?’* (**clinical pharmacist 2, FG2, Netherlands)**  Postpartum  *‘I would like information about doses post-partum, in the days following the delivery, where you have huge changes.’* **(psychiatrist 2, FG4, Netherlands)**  *I am very happy with this initiative (…) but I would prefer it if at some point, we didn’t only look at the pregnant woman and the fetus, or the neonate separately, but if we were able to fully describe the physiology of both mother and child around the delivery.’* (**clinical** **pharmacist 5, INT4, Netherlands)**  Fetal exposure  *‘This makes me wonder: what additional knowledge could we generate, especially regarding fetal exposure?’* (**anaesthetist 3, INT2, Netherlands)**  Fetal safety  ‘*I can imagine that the model tells you very little about teratology.’* (**gynaecologist 1, FG6, Netherlands)**  *‘I'd say that the longer term outcomes for baby are one of the biggest areas that women want to know about (…). And so, I think it doesn't go all the way to kind of answer one of the main questions that a majority of women have.* **(midwife 3, INT13, UK)**  Model-informed doses as additional evidence  **‘***I find it very promising that you could use a model instead of clinical data in order to give well substantiated advice.’* **(general practitioner 2, FG3, Netherlands)**  *‘I would follow MIDs because there is probably no better information available out there.’* **(anaesthetist 1, FG1, Netherlands)**  On whether she would follow recommendations from : *‘Yes for sure. It would*  *give us something to rely on. (…) Many of the anaesthetics for (…) pregnant women are used off-label. (…) This way we could prescribe medication based on an actual rationale.***’ (anaesthetist 3, INT2, Netherlands)**  *‘The model gives you more information than you currently have so it is helpful.’* **(clinical pharmacist 3, FG2, Netherlands)**  ‘*Sometimes you have information on effectiveness, for example with anti-epileptics where you know that enzyme induction leads to reduced exposure and more frequent insults (during pregnancy). You could use PBPK models as an additional piece of evidence showing that reduced effectiveness comes alongside reduced exposure. (…) This way we could generate more solid recommendations. This would work as a kind of external validation.’* **(clinical pharmacist 5, INT4, Netherlands)**  *‘I am keen on the idea of a pregnancy formulary, even for a small number of drugs (…). At this stage, there is no information whatsoever. So for those medications that have been well-researched, some guidance would be of great added value.***’ (clinical pharmacist 2, FG2, Netherlands)**  *‘We currently prescribe antibiotics despite them not having been researched in pregnant women. So it’s not like we can say with 100% certainty that we are doing the right thing. So if* (MIPF dose recommendations) *are backed by evidence, we should follow them because it has been better researched than what we have done so far.’* **(gynaecologist 12, FG12, Netherlands)**  *‘The numbers we use aren't evidence-based. So actually, I don't really see how the data can be inferior to what we're currently doing. And I hope it was more informed than superior to it.’* **(obstetric physician 1, INT9, UK)**  *‘If you pull it off, then it will be a great addition to what we already have*. *It definitely would support a lot of the limited data that's out there already*.’ **(midwife 4, INT13, UK)**  *‘As long as it is safe and accurate, it's probably better than nothing. Rather than just saying no.’* (**obstetric physician 3, INT13, UK)**  Risk of a MIPF: the need for interpretation and a critical use  *‘The risk of a formulary is (…) that people with less knowledge apply (dose recommendations) as if they were the truth and don’t adjust them for individual cases but simply as ‘I am seeing a pregnant woman, I consult the formulary and this is the dose.’* **(clinical pharmacist 3, FG2, Netherlands)**  *‘Paediatricians in regional hospitals follow the paediatric formulary blindly.’* **(clinical pharmacist 4, in training, FG2, Netherlands)** |
|  |  |  |
|  | Knowledge generation | *‘It is a really sensible way of trying to expand the knowledge that we have.*’ (**clinical pharmacist, INT7, UK)**  Participation of pregnant women in research  *‘This is wat stops people from participating in research (…) What goes to the baby and what does it do with the baby’s development? (…) If you know more* *from a model, you can give better answers to these questions*.**’ (midwife 3, FG7, Netherlands)**  *‘If we can draw on the knowledge from the Western world on (…) dose adjustments (…), as we start to use those drugs* (for noncommunicable diseases). *We're coming into it with a position of knowledge from the mistakes made elsewhere and maybe we can develop something better.’* **(internist 3,* FG8, Uganda)**  *‘I think it's really, really amazing, really, really wonderful to take away the risks from the mothers and actually be able to do this in the lab. I think it will accelerate a lot of things.’* (**clinical researcher, FG8, Uganda**)  Subgroups of pregnant women  *‘I see a lot of potential for it even globally if you think about it because if you can get data from different sources then it's not just going to be that for example Africa is waiting for the information that's coming from the Western world. It may also then start to show differences in how medications are processed by different populations.’*  (**gynaecologist 11, INT11, Kenya)**  *‘I think it opens up an opportunity for countries such as Kenya to be involved in this kind of work.* **(gynaecologist 11, INT11, Kenya)**  *‘It would be very convenient to test* (a drug of interest) *in an appropriate population, because if this same drug will be used, say, in low income countries say in Africa, it will also be appropriate to get some data regarding that drug in this population’* (**research pharmacologist, FG10, Uganda)** |
|  | Selection of medications | Most frequently used  *‘SSRIs which are very widely used. This would be the first choice for psychiatry.’* **(psychiatrist 2, FG4, Netherlands)**  *‘Psychotropics considering how many people use them’* **(gynaecologist 2, FG6, Netherlands)**  Other considerations  *‘I would be more interested in medication that you start during the pregnancy (…) for example antibiotics.’* (**gynaecologist 3, FG6, Netherlands)**  ‘*The largest gains can be made by using the model for medications for which there is very little data.*’ (**internist 1, in training, FG4, Netherlands)**  *‘Medications that have potentially toxic effects.’*  (**gynaecologist 3, FG6, Netherlands)**  *‘Drugs for which you have no clinical feedback.’* (**gynaecologist 3, FG6, Netherlands)**  *‘I don't know how the priority of inclusion of drugs would be since most of the work may be done in Europe, with having a variety of people in the panel who can also suggest that. There may be some drugs from Africa or more commonly used in other countries*.’ (**clinical researcher, FG8, Uganda)**  *‘We can access a lot of medications over the counter, much more than (…) in the United Kingdom.’* **(gynaecologist 11, INT11, Kenya)**  *‘Address the things that cause the highest maternal mortality and morbidity.’* **(gynaecologist 11, INT11, Kenya)**  *‘I think the priority should be based on the prevalence of conditions and that depends on where we are. The three main conditions responsible for maternal mortality and morbidity.’* **(gynaecologist 10, INT10, Cameroon)**  *‘It should be medications that we suspect do alter significantly in pregnancy, like the new anti-epileptic medications I think would be useful.’* **(obstetric physician 2, INT12, UK)**  *‘So if I was doing this, I'd look at actually what your quick wins and what's the biggest cause of problems in your region. So if it was me in the UK, the leading cause of is due to mental health conditions, cardiac disease, neurological disease*.’ (**obstetric physician 3, INT13, UK)** |
| Complexity |  | ‘*This is very complicated.’* **(general practitioner 4, FG3, Netherlands)**  *‘It sounds very complicated to me given the many ways in which medications can be administered.’* (**midwife 3, FG7, Netherlands)**  *‘For me this is still a bit abstract because I then think, OK but which data are you going to input into the model?’* (**psychiatrist 1, FG1, Netherlands)**  *‘This is hard to grasp.’* **(community pharmacist 2, FG5, Netherlands)**  **‘***This is a topic that* (requires expertise) *across specialties*, (…) *this makes it difficult for many people*.’ (**neurologist, INT1, Netherlands)**  *‘How much can we truly rely on (the modelling) when we are counseling women and their families? I don't know without interrogating your modelling, which, quite frankly, will probably give me brain ache, because getting your head around that is very, very difficult.’* **(obstetric physician 3, INT13, UK)**  *‘And then kind of translating that I guess, into sort of more layman's terms to properly counsel women, I think will be incredibly challenging.’* **(midwife 4, INT13, UK)**  *It's promising, but it's (…) such a huge body of work and there are so many variables here, let's say for example, women with chronic kidney disease. The change in their renal function is significantly going to affect the pharmacokinetics, pharmacodynamics of certain medications. And ethnicity. (…)* Also: *It's not just (…) the transfer to the fetus and the short term implications with regards to teratogenicity, it's long term.* *(…) I appreciate your modelling is not going to be the full answer. But I think you may find that it is going to be very complex.’* **(obstetric physician 3, INT3, UK)** |
| Credibility | Model parameters & applicability | ‘*You'll think hard about it if the dose suggestion was quite out width what I had done previously or outside the normal ranges for non-pregnant*.’ (**obstetric physician 2, INT12, UK**)  Input parameters  *‘How is the metabolism of a drug by each organ system determined?***’ (gynaecologist 2, FG6, Netherlands)**  *‘How do you correct for the interindividual variability between placentas?’* (**gynaecologist 2, FG6, Netherlands)**  *‘I feel like blood concentrations do not always reflect well what happens within tissues.’* **(gynaecologist 1, FG6, Netherlands)**  *What for influence does a child’s size have over the course of the pregnancy? I feel like this cannot be investigated. (…) All in all I feel like where do you even begin*?’ (**midwife 3, FG7, Netherlands)**  *‘ A model is just as good as information you put into it, right and it's just a tool to help us.’* **(clinical pharmacologist, INT16, South Africa)**  Applicability  *‘So my question is, is it individualized per patient?* ***(*gynaecologist in training 8, FG9, South Africa)**  *‘If you are taking all the time to model such physiological changes and it's based on someone with a normal BMI, then in the South African contexts I don't even know how accurate the doses would be because. We have so many people with a BMI of 40’* **(junior doctor, FG9 South Africa)**  *If it was (…) almost population specific, so that you actually are trying to create a platform for specifically the South African population* *where they take HIV into consideration, the obesity, the different cultures (…) like for example in the Indian population, there's definitely a high prevalence of diabetics (…).* *And I think that that's probably what the model can do, right. It can be population specific*.’ ***(*gynaecologist in training 8, FG9 South Africa)**  *‘I would like to have a system where you can input data on a pregnant like her weight, how fit she is, if she has conditions like preeclampsia and IUGR (…) and get a dose range.’* (**gynaecologist 9, INT8, UK)** |
|  | Validation | ‘*I am skeptical about models that haven’t been clinically validated and would not want to blindly apply a model-informed dose in that case.’* **(clinical** **pharmacist 1, FG2, Netherlands)**  ‘*How can you validate* (your model) *in the first and second trimester? (…) There are medications that are safe in the third trimester but which aren’t in the first trimester.*’ (**general practitioner 4, FG3, Netherlands)**  ‘*Although some clinical validation is always helpful, I think that as models demonstrate that their predictions are accurate more and more frequently, that the acceptability of their predictions will be greater.’* **(gynaecologist 3, FG6, Netherlands)**  *‘While I trust computer algorithms (…) I would like to see that it actually works in practice.***’ (community pharmacist 2, FG5, Netherlands)**    *‘Taking twice as many antibiotics (… ) may make sense for treating the infection but (…) in the absence of clinical experience, what does it mean for the fetus? You said that there is limited clinical data to assess the model. I find this very concerning.’* **(psychiatrist, FG12, Netherlands)**  ‘*I would need time to verify this in practice. I would want the feedback of monthly blood concentrations and patient conversations and then compare the outcomes (…) so I get a personal feel for this.’* **(neurologist, INT1, Netherlands)**  *‘I find the model super interesting but it’d be good to know how carefully this has been thought through so I can tell (women) with a hand on my heart that this has been very well researched.’* **(midwife 3, FG7, Netherlands)**  *‘So we are going to have a preliminary dose because of the modeling. But then is there anything in place to further close the loop by giving the dose, taking samples or testing concentrations and inputting them back*.’ (**clinical pharmacologist, INT16, South Africa)**  *‘The validity of the model is a concern, (…) I would want to be assured that it had been validated.’* (**clinical pharmacist, INT7, UK)**  *‘I’m happy to work with MIDs but I would need evidence points that validate the model. I wonder: what is the number of real world observations that are considered to be sufficient to validate the model? This must be defined for other populations*.’ (**gynaecologist 9, INT8, UK**)  The need for local validation  *‘Policy making and guideline making boards (…) would need to see some local validation data. So I think most countries, say if you did a validation in the Netherlands, we'd probably have to repeat it in the UK or they'd have to repeat it in India for them to actually think it's applicable to their population even though the Netherlands and UK are really similar.’* (**gynaecologist 7, INT6, Vietnam, India, UK)**  *If (…) a drug that is (…) eliminated by an enzyme that is highly polymorphic, and now it's even in pregnant women, then you would really want evidence in a particular population, at least in which it is intended to be used.*’ **(clinical pharmacist 6, FG10, Uganda)**  *‘What is it based on and can we truly rely upon the modelling? Where there is a paucity of data, it'll be quite difficult to work out if the modeling is correct. You know, you can rely upon the data you have, even if it's limited with some respects. Obviously, you have to interrogate how that data was collected and any report by us or any biases within that. (…) But what do we really know about the modelling and where are the potential issues and biases?’* (**obstetric physician 3, INT13, UK**) |
|  | Institutional endorsement | *‘I would trust a model-informed formulary like I trust the Dutch paediatric formulary. Perhaps this is a bit naive but if all this information is available online and official I would follow it.’* (**anaesthetist 1, FG1, Netherlands)**  *‘If you know that it's approved by a South African organisation because I wouldn't want to get in trouble for using a European recommendation and South African obstetricians have reviewed and approved it for use.’* **(junior doctor 2, FG9, South Africa)**  *‘Let's say this has been passed by Minister of Health or by the National Drug Authority. (…) Once it's policy or it's a guideline,* (HCPs in the field) *will always be able to prescribe this medication.’* **(clinical researcher, FG10, Uganda)**  *‘The other thing a NICE committee that always sways the balance: What's the cost implication of doing this or not doing this?’* **(gynaecologist 7, INT6, Vietnam, India and the UK)** |
| Feasibility |  | *‘Saying it’s a massive piece of work is an understatement, isn't it?’* **(midwife 3, INT13, UK)**  Model parameterisation  *‘Pregnancy is a dynamic process. Your renal clearance, for instance, doesn’t change overnight. How do you approach this over time?’* (**anaesthetist 1, FG1, Netherlands)**  *‘It sounds very time consuming to collect all the input data needed for the models.’* **(general practitioner 1, FG3, Netherlands)**  ‘*The (human) data will be scarce. Then you might end up relying on pharmacovigilance (…) data only.’* **(gynaecologist 10, INT10, Cameroon)**  Model verification  *‘How do you verify this? It looks very difficult to me.*’ (**midwife 3, FG7, Netherlands)**  Selection of medications  ‘*I would start with low-hanging fruit. If there are studies available for medications with a known target, then you don’t need to do a lot of extra work (…) and convince people.***’ (clinical pharmacist 4, in training, FG2, Netherlands)**  Therapeutic range  *‘At least for lamotrigine we know that there are very large intra-individual differences so I was wondering: should you not integrate pharmacogenetics in your models?’* **(neurologist, INT1, Netherlands)**  *‘Concentration-effect relationships are already complex in a non-pregnant population, never mind in pregnant women.’* (**clinical** **pharmacist 3, FG2, Netherlands)**  *‘There is a large interindividual variation in blood concentrations for a given dose, independently of the effectiveness. This looks like it would be hard to include in the model, unless you have baseline information of the individual.’* **(psychiatrist 2, FG4, Netherlands)**  *‘Ideally you would want to do some blood sampling and check what happens* (during pregnancy). *(…) It makes a difference whether you want that a medication remains at the same concentration, or whether it can fluctuate a bit like anti-epileptic drugs. Some medications, on the other hand, have a lower threshold* (of effectiveness) and we know that there is a lot of variation in how quickly women reach that lower threshold*.’* **(neurologist, INT1, Netherlands)**  *‘This debate comes up a lot like which exposure are you aiming for. Should you just presume you’re aiming for the same exposure as an adult and does that translate into efficacy if you have a good exposure to the drug, like is it actually goina result in treatment success in the end, without it being unsafe.’* (**research physician, INT16, South Africa**)  Formulations  *‘Even common drugs will be coming in slightly different dosages just because of the way the formulary is working in those countries*.’ **(gynaecologist 7, INT6, Vietnam, India,UK)**  *‘There's only so much dosing or adjustment dosing that is possible to do isn't there? I guess you got to work within that limitation.’* **(gynaecologist 7, INT6, Vietnam, India, UK)** |

2. USERS

| Awareness |  | *‘We know way too little about medication doses in pregnancy and medication use in general.’* (gynaecologist 2, FG6, Netherlands)  ‘C*hildren and pregnant women are often excluded from research on those* (anaesthetics) *(…) meaning we use them off-label*.’ (anaesthetist 3, INT2, Netherlands)  *‘There is a critical need for evidence-based dosing advice (in vulnerable populations).’* (clinical pharmacist 3, FG2, Netherlands)  *‘You cannot do much about it* (doses in pregnancy), *which actually goes fine in most cases. For a lot of medication it appears to be unnecessary*.’ (general practitioner 3, FG3, Netherlands)  *‘You don’t have to make changes in the medication doses of a pregnant woman very often. It isn’t a very complex question as far as I am concerned.’* (general practitioner 2, Netherlands)  With epilepsy: ‘*you instantly get this feedback of wait, you are doing this wrong (with medication dosing) because there are still insults. And that is probably different with other conditions.’* (neurologist, INT1, Netherlands)  *‘We are very cautious when choosing drug doses in pregnancy, it’s likely that we frequently underdose certain medications.’* (gynaecologist 6 and clinical pharmacologist, FG6, Netherlands)  *‘There’s little attention given to the fact that when someone is pregnant, changes in the medication dose might be needed. (…) Neurologists just tell patients that they should stop e.g. their migraine medication.’* (neurologist, INT1, Netherlands) |
| --- | --- | --- |
| Knowledge |  | Pharmacology  *‘Pharmacology is a neglected area in the medical curriculum.’* **(gynaecologist 6,* FG6, Netherlands)**  **‘(**Medication use in pregnancy) *is a very specific area of expertise****.’* (psychiatrist 2, FG4, Netherlands)**  *‘I am just thinking back on my general ignorance of pharmacokinetics. I mean literally I think there are a lot of doctors out there who are like what does PK stand for.’* (**research physician, INT16, South Africa)**  Pharmacokinetics/medication dosing in pregnancy  *‘I admit I don’t know much about (…) dosing in pregnancy.’* (**general practitioner 1, FG3, Netherlands)**  *‘We know what kind of huge physiological changes take place* (in pregnancy).***’* (clinical** **pharmacist 1, FG2, Netherlands)**  *‘We know that the blood levels of several anti-epileptic drugs dramatically decrease during pregnancy and that this must be addressed. (…) Another thing to consider is whether a decrease under a given threshold also has an influence on the frequency of insults. This has long been known for lamotrigine for example which is why we monitor the blood concentration (…) in the pregnancy.* (**neurologist, INT1, Netherlands)**  *‘We normally dose medications based on length and weight but we know that for example the metabolism, the distribution volume and the heart-minute volume of* (pregnant) *women are different. If you think of (…) an area under the curve it’s likely that we are underdosing slightly but at the same time we can’t just rely on an arbitrarily chosen higher or lower weight.’* (**internist 2, FG4, Netherlands)**  *‘At some point, we had four pregnant women with psychiatric conditions in one year. That definitely made us panic a bit*.’ **(community pharmacist 2, FG5, Netherlands)**  Unfamiliarity with pharmacokinetic models  *‘It looks impressive. (…) We have little feeling for models (…). I don’t really know how* (the models) *are built and of course I do really trust you but still (…) the fact that I don’t really know how it works and how it is measured, this makes me feel uncertain.’* **(community pharmacist 1, FG5, Netherlands)**  *‘We did not learn about (*pharmacokinetic models*) during our training, which makes it hard to grasp for us.’* **(community pharmacist 2, FG5, Netherlands)** |
| Attitude | Reference framework | Medication use and dosing in pregnancy (HCPs)  *‘**In practice, most* (HCPs) *have this sense of ‘do no harm’, meaning that you are more focused on whether the drug is safe.’* (**clinical** **pharmacist 3, FG2, Netherlands)**  *‘I think that like most people I am conservative with regards to medication use in pregnancy because I have to.’* (**general practitioner 3, FG3, Netherlands)**    *‘I am generally quite defensive when it comes to prescribing medication during a pregnancy (..). Better safe than sorry is my creed.’* ***(*general practitioner 2, FG3, Netherlands)**  *‘As doctors we will always err on the side of caution when it comes to medication doses in pregnancy.’* (**gynaecologist 5, FG6, Netherlands)**  *‘Safety prevails over dosing. Doses are a prolongation of (fetal safety), that is, which dose is safe?***’** **(general practitioner 3, FG3, Netherlands)**  *‘I feel like* (pharmacological care in pregnancy) *sometimes is suboptimal for women. I think that (…) their healthcare needs are not prioritized over the risk to the baby*.’ (**clinical pharmacist, INT7, UK)**  ‘*Generally speaking physicians are quite risk averse when it comes to pharmacological treatment and that comes from a very good place, which is, you know, first do no harm.*’ **(obstetric physician 2, INT12, UK)**  Medication use and dosing in pregnancy (pregnant women)  *‘The first question of patients is generally: is it safe?’* **(general practitioner 2, FG3, Netherlands)**  *‘Patients that consult with m often want to take the lowest possible effective dose.’* **(internist 1, in training, FG4, Netherlands)**  *‘Patients are not at all concerned about the dose, they are just focusing on whether or not they can use the medication.***’ (gynaecologist 2, FG6, Netherlands)**  ‘*I don’t think that doses are something many patients think about much*.’ (**neurologist, INT1, Netherlands)**  *‘What I find difficult with SSRIs is that women phase them out themselves because they think ‘I am pregnant and I shouldn’t use this anymore’, and then they phase the SSRIs out without proper supervision.’* **(midwife 3, FG7, Netherlands)**  *I think it's useful, it's good for women to know what's going into their bodies. But it's nice not to overwhelm patients with a lot of information*.’ (**gynaecologist 11, INT11, Kenya)**  *‘They often don't worry about themselves so much. They just wanna know what the risk is to the baby.’* (**obstetric physician 2, INT12, UK)**  ‘*Pregnant women want the lowest effective dose*.’ **(gynaecologist 9, INT8, UK)**  ‘*On the whole a woman will think about the impact of anything on her baby first rather than her. It's a very emotive area we're talking about and we saw that very clearly with COVID-19 and vaccination*.’ **(obstetric physician 3, INT13, UK)**  Computer models  ‘*Computer algorithms are everywhere these days. So this must work.’* **(community pharmacist 2, FG5, Netherlands)**  ‘C*omputer models are generally very hard to understand*.’ (**gynaecologist 2, FG6, Netherlands)**  *I think that you're just scratching the surface of model-based dosing and I think this is the way we are going.* **(clinical pharmacologist, INT16, South Africa)** |
|  | Information needs | ‘*If I am to adequately advise patients or doctors, I must fully understand how this works, I must get it 100%.’* **(community pharmacist 1, FG5, Netherlands)**  *‘We must know how it works and what it’s based on to be properly informed for discussions with colleagues.’* **(community pharmacist 2, FG5, Netherlands)**  *‘I would like to know how the model works and what parameters are taken into account.’* **(gastroenterologist, INT3, Netherlands)**  ‘*I currently follow dose recommendations from the Dutch formulary with little knowledge of how these recommendations came to be.* (**general practitioner 1, FG3, Netherlands)**  ‘I*t doesn’t really matter to me whether the information comes from computer models or from another source. I am (…) quite pragmatic in this regard.***’ (general practitioner 1, FG3, Netherlands)**  ‘*I trust the advice of professionals that know about the subject.’* (**general practitioner 2, FG3, Netherlands)**  *‘I don’t need to know about the individual equations of the model (…) but I’d be very interested to know about the assumptions behind those equations and how they are linked to physiological parameters.’* (**anaesthetist 1, FG1, Netherlands)**  *‘I want to understand how the advice was generated so I can explain to my pregnant clients that their dose should be adjusted because of changes in efficacy and what we know about a child’s exposure to the medicine- so I can tell them that there is evidence to support dose adjustments.’ (***midwife 3, FG7, Netherlands)**  ‘*I just think people trying to understand the background as to how these dosing recommendations came about might be quite specific to the people familiar with pharmacometrics*.’ **(clinical pharmacologist, INT16, South Africa)** |
|  | Willingness to change | Of HCPs  *‘I would be very much inclined to consult this resource and take the dose recommendations into account.’* (**anaesthetist 1, FG1, Netherlands)**  *‘The old guard will be less inclined to change.’* (**anaesthetist 2, FG1, Netherlands)**  Of pregnant women  ‘*If pregnant women see that the dose must be increased (…) they will worry: can this be harmful for my child?*’ **(midwife 3, FG7, Netherlands)** |
|  | Shared decision-making | ‘*Some patients will be sufficiently informed by being told that there is a specific formulary for pregnant women that underlies their dose. The dose won’t be that important for most patients as long as you can tell them that is based on something. It is all very individual.***’ (gynaecologist 3, FG6, )**  *‘People who have been using medication for a long time can interpret* *exposure. They will know that if the child is exposed to 10% of the dose that they use, that’s not very much.’* **(community pharmacist 1, FG5, Netherlands)**  *‘I find it challenging to explain something complex in simple terms* (to patients)*.’* (**general practitioner 2, FG3, Netherlands)**  *‘Whether or not you can use shared decision-making depends on the patient. There are patients for whom it doesn’t make sense at all but there’s also people who want to think the decision through (…). You’d give more detailed explanations to these people.’* **(community pharmacist 1, FG5, Netherlands)**  *‘If I tell a woman you should take this pill three times a day, she will definitely not ask why it’s three times instead of twice a day.* (…) *The only thing she might ask is, can this harm my baby, and if the answer is no, the conversation ends there for most women.’* (**gynaecologist 12, FG12, Netherlands)**  *‘Information about models is too complex for patients.’* **(gastroenterologist, INT3, Netherlands)**  ***‘****I would like to discuss information about models with patients, I don’t tend to hold any secrets for them regarding my considerations*.’ **(general practitioner 2,** **FG3, Netherlands)**  *‘I wouldn’t try to fully understand the models and would just openly tell patients that this is the best knowledge that we have and that it’s not 100% foolproof, like everything else in healthcare.*’ **(general practitioner 3, FG3, Netherlands)**  *‘The culture is that the women have a lot of faith in trust in the doctors, so it's unlikely that a woman would protest or (…) ask why certain medications certain doses have been given.’* **(gynecologist 11, INT11, Kenya)**  *‘I don't think pregnant women are particularly worried about dosing because ultimately I don't think they're in a position to understand the benefits of changes in dose and the nuances of the scale of dosing.’* **(obstetric physician 1, INT9, UK)**  *‘Most women don’t know that drugs in pregnancy are used off-label and that there is no evidence for it. They would be horrified if they knew. Most doctors don’t tell patients that they are getting off-label drugs even though they should.’* **(gynaecologist 9, INT8, UK)**  *‘I think we need to empower women and give them the information and not hide it from them.*’ (**obstetric physician 3, INT13, UK**)  *‘I see a high proportion of nonwhite patients (…). For some groups of women, there's almost an expectation for me to be a bit paternalistic, because that's the cultural norm that they are used to. (…) I’m there to do what the doctor says and they’re the expert and they're going to take the decisions. I don't want that weight of decision or burden and guilt on me. I'm going to do what I am told because I'm told it's the best thing.’* (**obstetric physician 3, INT13, UK**) |
| Behaviour | Work routine (prescribing & dosing practices) | Dosing practices  **- One-size-fits all**  ‘*We pretty much always apply the dose recommendations that are used outside of pregnancy*.’ **(gynaecologist 3, FG6, Netherlands)**  *‘It is all about switching medication, not really about adjusting the dose.’* **(general practitioner 1, FG3, Netherlands)**  ‘*We have clear dose recommendations for anaesthetics in adults and follow these recommendations for pregnant women*.’ (**anaesthetist 3, INT2, Netherlands)**  *‘A significant number of women need antibiotics at some point of their pregnancy but we act as if nothing changes* (when dosing*).’* **(clinical** **pharmacist 1, FG2, Netherlands)**  *‘It’s really standardized dosages. It is very unusual that we tailor them.’* **(gynaecologist 11, INT11, Kenya)**  *‘There's always just the assumption that the dose is the same as in any other adult dose.’* (**clinical pharmacist, INT7, UK)**  **- Or a pragmatic approach**  *Often we don’t know and we just figure something out (…) based on our clinical experience***.’ (clinical** **pharmacist 1, FG2, Netherlands)**  *‘I haven’t really encountered medication for which I struggled to determine the appropriate dose in pregnancy because there generally is time to adjust the dose based on a patient’s symptoms or blood concentration of the drug.’* **(internist 1, in training, FG4, Netherlands)**  *‘Dosing occurs based on clinical experience, but this clinical experience, of course, is not available in many places.’* **(psychiatrist 2, FG4, Netherlands)**  *‘Pregnant women who use nifedipine once daily tend to have an elevated blood pressure (…) much more often than women outside of pregnancy. Because of this, I tend to dose pregnant women twice a day.’* (**internist 1, in training, FG4, Netherlands)**  *‘For many of the medications that we use, we notice that the effect can change during pregnancy. In that case, we adjust the dose and wait to see whether this leads to the desired result.’* **(psychiatrist 2, FG4, Netherlands)**  *‘We are quite used to sort of dosing of experience now for the complicated medication.(…) In that situation I'd rather involve renal physician or an internal medic rather than adjusting the doses myself.’* (**gynaecologist 11, INT11, Kenya)**  *‘We make extrapolations (…) from the non-pregnant population and it's feels sometimes like a best guess.’* **(obstetric physician 1, INT9, UK)**  **- Exception: pregnancy adjusted-doses**  *‘We have agreed on certain doses* (in pregnancy) *in a number of protocols e.g. the hypertension protocol.’* (**gynaecologist 5, FG6, Netherlands)**  *‘Our protocol says that you should monitor the blood levels of lamotrigine. Then came oxcarbazepine. It currently says that we don’t know for levetiracetam.’* **(neurologist, INT1, Netherlands)**  *‘There are virtually no drugs apart from a few thyroid treatments for which men will start monitoring proactively and adjust the dose because you know that there will be changes in the concentration whose consequences are considered significant*.*’* (**clinical** **pharmacist 5, INT4, Netherlands)**  *‘I'm also very lucky because I'm networked with other obstetric physicians. If there's something I don't know, I'll often ask my colleagues (…) and someone's goina know the answer.’* **(obstetric physician 3, INT13, UK)**  Information sources  *‘What we use is the national formulary, anesthesiologic books as well as PubMed for drugs that we use less often.***’ (anaesthetist 3, INT2, Netherlands)**  *‘When we have to look up something we use the Dutch TIS*.’ (**midwife 3, FG7, Netherlands)**  *‘If the information in the Dutch TIS is not clear, I will just have a look at the academic literature.***’ (clinical** **pharmacist 1, FG2, Netherlands)**  *‘I must admit that I don’t often look at the Dutch TIS.’* **(general practitioner 2, FG3, Netherlands)**  *‘I knew that there was a teratology information service but I didn’t know that you could find their information online, this is really new for me.’* **(community pharmacist 2, FG5, Netherlands)**  *‘I mostly look at the Dutch formulary.’* **(general practitioner 2, FG3, Netherlands)** |
|  | Work routine (shared decision-making) | *‘I discuss (*medication doses in pregnancy) *with patients.’* **(internist 1, in training, FG4, Netherlands)**  *‘I will tell (patients) what the average dose is and what the maximum dose is but they know it’s also about how they tolerate this (…) and about remaining seizure free.’* **(neurologist, INT1, Netherlands)**  *The problem (…) is that we are so overwhelmed with our clinic numbers, you don't actually have time’* (**gynaecologist in training 8, FG9, South Africa)**  ‘*Certainly in Vietnam, the clinics were so busy, I don't know how much time there would be for that sort of counseling*.’ (**gynaecologist 7, INT6, Vietnam, India, UK)** |

1. SOCIAL AND ORGANISATIONAL FACTORS

| Organisation |  | Interdisciplinary care  *‘Patients that (…) have a chronic disease like diabetes or inflammatory bowel disease are followed up in the hospital where a strategy regarding (*medication use in pregnancy) *has often already been discussed.’* (general practitioner 5, FG3, Netherlands)  *‘We are quick to confer with pharmacists.*’ (general practitioner 2, FG3, Netherlands)  *‘I think the way maternity care tends to work around the world has to be very simplistic to deliver because the level of training of the health workers is probably not at a level where they're discriminating different doses.*’ (gynaecologist 7, INT6, Vietnam, India and the UK)  Local initiatives to improve pharmacological care in pregnancy  *‘Hospital X started a medication advisory point for pregnant women. (…) We also wanted a kind of regional expert centre primarily (…) where complex patients or pregnant women who use medication can be counselled.’* (clinical pharmacist 3, FG2, Netherlands)  ‘*There’s no national guideline for* (*TDM) of medications for intestinal bowel disease during pregnancy (…) but we have a local guideline that I wrote*.’ (gastroenterologist, INT3, Netherlands)  *‘Four years ago, I set up an outpatient clinic for patients with Crohn and colitis that want to undergo a medically supervised pregnancy (…). It’s preconceptional care and (…) I do this together with two gynaecologists.(…).’* (gastroenterologist, INT3, Netherlands)  *‘The last ten years we have developed a care pathway for women* *with a pregnancy wish and epilepsy. The focus lies on providing the best possible counselling in pregnancy (…) including advice on (…) medication use (…) and trying to optimize pregnant women’s medication regimen. (…)’* (neurologist, INT1, Netherlands)    *‘Counselling and blood sampling* (of pregnant women using anti-epileptic medication) *is done by specialist nurses* (…) *whom* *I supervise (…) on behalf of my colleagues based on my expertise of this topic. (…) So you can see that this* (type of care) *is not well institutionalized, which I find problematic.’* (neurologist, INT1, Netherlands)  The role of clinical guidance  *‘I don't know if they have formal written guidelines (…), but it's just more that’s what they do. They learn from the apprenticeship model*.’ (gynaecologist 7, INT6, Vietnam, India, UK)  *‘In the Kenyan context, it's a lot about what we've been taught in school. in the Kenyan context, it's a lot about what we've been taught in school. in the Kenyan context, it's a lot about what we've been taught in school.’* (gynaecologist 11, INT11, Kenya) |
| --- | --- | --- |
|  |  |  |
| Culture |  | Dosing in pregnancy: a blind corner  *‘The world has been designed for the standard reference male. We really don’t look at differences but if you were to look, you would spot other significant risks*.’ (**clinical** **pharmacist 1, FG2, Netherlands)**  *‘I'm not really aware of many drugs where we know that we need to change the dose, but that's also worrying. (…) I worry that sometimes the question has just not been asked.’* **(research clinician, FG18, Uganda)**  *‘Some of the recommendations I’m quite blind and I don't think you really think about that until you see alternative ways in which you could make recommendations. You're kind of like, well, this is just the way it is.’* (**clinical pharmacist, INT7, UK)**  Fetal health  **- A societal priority**  ***‘****I think* (pregnant women) *would be very interested in this kind of information because across the world really a baby is precious. You want your baby to be fine. You know, it's both at the personal level and societal level.’* (**clinical researcher, FG8, Uganda**)  **- Do no harm**  *‘I think a lot of it comes from our culture of do no harm first and medication is seen as harm.’* (**midwife, INT3, UK)**  Decision-making under higher uncertainty  ‘*A lot of what we do we do with very, very minimal data*.’ (**obstetric physician 3, INT13, UK)**  Shared decision-making  *‘Shared decision making (…) is the approach that is culturally accepted by most clinicians in the UK.’* **(obstetric physician 3, INT3, UK)**  Universality of clinical guidance  *‘There is a perception that these (WHO) global guidelines may not apply to their population, like a population exceptionalism.*’ **(gynaecologist 7, INT6, Vietnam, India,UK)** |
| Legality |  | *‘I suppose I'm talking about (…) the legal fears. Unless you could show that was a reasonable thing to do and that other clinicians in your situation would have done exactly the same thing then you are personally responsible for that outcome, like a legal, but also in an ethical and moral way. I suppose those are the things that I would be thinking about, like some kind of assurance that what I'm doing is the right thing to do.’* (**clinical pharmacist, INT7, UK)**  *‘Obviously there was always going to be medically legal implications for these things and therefore getting the trust (*to approve the MIPF is key).*’* **(obstetric physican 2, INT12, UK)** |

4. IMPLEMENTATION PROCESS

| Awareness-raising and education | Overall strategies for awareness raising and education | Target audiences  *‘You are developing (something) for a very diverse group.’* (neurologist, INT1, Netherlands)  ‘*The dissemination of information (…) is always difficult because there is such a spectrum of people you’re trying to reach. And that is both in medical specialty as well as geography.’* (obstetric physician 1, INT9, UK)  Communication  ‘*Getting something to become part of the routine of all general practitioners takes time and effort. But a good story helps*.’ (general practitioner 2, FG3, Netherlands)  *‘The Dutch Paediatric Formulary is used very widely which can help make people enthusiastic about the pregnancy formulary.’* (community pharmacist 1, FG5, Netherlands)  *‘I think it’d be good to raise awareness on the pregnancy formulary among your targeted audiences. One helpful strategy is to make the connection with the Dutch Paediatric Formulary.‘* (anaesthetist 3, INT2, Netherlands)  Proof-of-concept doses  ‘*Examples of* (MID recommendations*) would make the model more understandable*.’ (community pharmacist 3, FG5, Netherlands)  *‘PBPK, people or clinicians don’t know what it is. So (…)I think something to prove that this works helps close that loop and then it’s further evidence (…) to say listen this is what we’ve done for at least one or two drugs.’* (clinical pharmacologist, INT16, South Africa)  Selection of medicines  *‘Anti-emetics are also interesting if you are seeking to disseminate the use of the MIPF across the Netherlands because they are used in all hospitals.’* (gynaecologist 6, FG6, Netherlands) |
| --- | --- | --- |
|  | Dissemi-nation channels | Academic curriculums and centres  *‘I think you should definitely start with* (raising awareness*) as part of t*he midwifery curriculum.**’ (midwife 3, FG7, Netherlands)**  *‘I think it would have to go via universities (…). But if something is backed by the university then they can sort of infiltrate that into their protocols and into their practicing clinicians.’* (**junior doctor, FG9, South Africa)**    Journals, professional associations and congresses  *‘Get different professional associations involved to convince doctors and pharmacists to use* (the MIPF)*.’* **(community pharmacist 2, FG7, Netherlands)**  *‘You’ll obviously have to do some marketing (…). I think that it’d be interesting (…) to introduce the* (MIPF) *and how it came about at anaesthesiologic congresses.*’ (**anaesthetist 3, INT2, Netherlands)**    *‘Publish information on the formulary in journals, for example the journal of psychiatry.’* **(psychiatrist 2, FG4, Netherlands)**  *‘I think you should definitely get the professional associations involved and arrange trainings for them.’* **(midwife 3, FG5, Netherlands)**  ‘*When you launch, you can raise awareness (…) using the newsletter of the KNMP (the Royal Dutch Society of Pharmacists*).’ **(community pharmacist 1, FG5, Netherlands)**  **‘***You could think of the NTvG (*Dutch Journal for Medicine). *I believe that pretty much all doctors read it*.’ **(neurologist, INT1, Netherlands)**  *‘If you want to disseminate this, I would think that the major obstetrics and gynecology groups and societies within different countries. I know the obstetricians focus a lot on guidelines, so it will be good to just get in there.’* **(clinical pharmacologist, INT16, South Africa)**  Local and regional champions  *‘All new anti-epileptics were first used in epilepsy centres where experience could be gained. This experience could then be shared during congresses (…). This is probably the quickest way to get people to* use (an innovation) *and hopefully to make them keen to explain how this works and how useful it is to others. (…)’* **(neurologist, INT1, Netherlands)**  ‘*Have a sort of ambassador in each centre, somebody who is part of the network and who can spread information*.’ (**internist 2, FG4, Netherlands)**  *‘I think we could gain a good understanding of this, and that we could contribute to the dissemination of model-informed dosing.’* **(community pharmacist 3, FG5**, **Netherlands)**  *‘Get the pharmacists onboard; they are the ones that inform doctors when dose adjustments must be made.’* (**gynaecologist 9, INT8, UK)** |
| Acceptability & usability | Issuance of dose recom-mendations | Evidence generation  ‘*Build a sort of forum or reference model that can be applied for specific data, which means effective international collaboration can be promoted.****’*** (**gynaecologist 3, FG6, Netherlands)**  *‘I’d be interested in (…) getting information on which PBPK models you have developed and which assumptions (…) for the physiology of a pregnant woman were used so that we can learn from each other and build on each other’s work.*’ (**clinical pharmacist 5, INT4, Netherlands)**  Approval of doses by multidisciplinary board  *‘Let clinical experts review the dose recommendations, including for potential changes in pharmacodynamics during pregnancy.’* **(clinical** **pharmacist 5, INT4, Netherlands)**  ‘*There must always be someone that interprets the model prediction so you have the complete picture*.’ (**clinical** **pharmacist 3, FG2, Netherlands)**  *‘I would want to be assured that (…) people that were much cleverer than me and that I trusted because I have some kind line of trust with them, had gone. Yes, these are OK to use.’* (**clinical pharmacist, INT7, UK)**  *‘It's a layer of quality assurance which is important. (…) That helps me in terms of trust.’* (**obstetric physician 2, INT12, UK)**  ‘*Somebody has got to make the decision*.’ (**gynaecologist 9, INT8, UK)**  Institutional endorsement  *‘Using the government routes and also the professional body route would work well.’* **(clinical researcher, FG8, Uganda)**  *‘Have a WHO representative on the board. I think that would make a huge difference, because you kind of telling them your plan from the beginning.’*  **(clinical pharmacologist, INT16, South Africa)**  *‘But if NICE haven’t endorsed it, then it's not going to be used across the country here.’* **(gynaecologist 7, INT6, Vietnam, India, UK)**  *‘Endorsement from royal colleges, specialist societies and then from bodies like MHRA would be massively helpful.’* (**obstetric physician 2, INT12, UK)** |
|  | Framing of dose recom-mendation | Access to evidence/assessment of uncertainty  *‘I think there's an element of setting up a trusted website source where the methodology is clear for people to understand and you're able to access that information if you need it. Often it would be something that I don't need.’* (**obstetric physician 2, INT12, UK)**  ‘*Interpretation of the (available information) on the safety and dosing of a medication should lead to a very clear answer so that (clinicians) (…) can immediately apply it*.’ (**clinical pharmacist 5, INT4, Netherlands)**  ***‘****A user, when looking for a medication dose, doesn’t have time to additionally read all sorts of background literature.’* **(clinical pharmacist 4, in training, FG2, Netherlands)**  *‘If I’m completely honest, if I look at my average colleague, she just wants to have a dosing advice. You can share a bit of information on the rationale but that won’t be users’ main focus.’* (**anaesthetist 3, INT2, Netherlands)**  Knowledge of reduced exposure to a drug in pregnancy ‘*doesn’t have to be directly translated in an adjusted dose (…), but there could be an alert that if you use the standard dose, the halved exposure could lead to reduced effectiveness*.**’ (clinical pharmacist 5, INT4, Netherlands)**  *‘It’d be helpful if the uncertainty* (of a model prediction) *can be quantified, both for the doctor and patient. (…) This way you can determine how heavily you let the model-informed advice count in your dosing strategy.’* (**psychiatrist 2, FG4, Netherlands)**  *‘Show what sort of evidence is available. Is there clinical data on safety and effectiveness, or is there only a PBPK model or a case series? These results can either be translated into a dose recommendation if a lot of information is available, or into a warning if there isn’t.‘* **(clinical pharmacist 5, INT4, Netherlands)**  *‘It’d be great if we were also willing to share more information when less evidence is available and give a clear description of the quality of the evidence and label. This way, you would at least give someone a better starting point when it comes to making their own assessment of the risks and benefits.’* **(clinical pharmacist 5, INT4, Netherlands)**  *‘I would like to have access to the risk-analysis that was conducted alongside the modelling (…). This might not be relevant for all MIPF users but I believe it can be relevant for pharmacists.’* (**clinical pharmacist 4, in training, FG2, Netherlands)**  *‘If I want to make a judgement of your recommendation: where can I find further information like however little information it is that you used to come to that conclusion? I would want a further reference.’* **(clinical pharmacologist 6, FG10, Uganda)**  *‘How strong, how binding or how do you grade the recommendation from the editorial board?’* **(gynaecologist 10, INT10, Cameroon)**  ‘Y*ou are also going to be developing data that's probably going to go against certain guidelines that are acknowledged and accepted. You just need to throw it out there and explain why this is different.*’ (**clinical pharmacologist, INT16, South Africa)**  Background information on pharmacokinetics in pregnancy  *‘I’d find it very valuable if I could access information on the elimination, maternal and fetal metabolism of a medication.’* (**anaesthetist 3, INT2, Netherlands)**  *‘I would find* (information) *on placental transfer very useful.’* (**anaesthetist 3, INT2, Netherlands)**  *‘**You might want to get information on the fetal exposure to a given drug so you can share this information with the mother.’* **(community pharmacist 1, FG5, Netherlands)**  ‘*For me it’s crucial to interpret which changes in basic physiological parameters (…) occur between the pregnancy and post-partum to know why we may have to dose more frequently*.’ **(internist 1, in training, FG4, Netherlands)**  *‘The transfer information is really helpful. (..) So actually, knowing that something really isn't transferred quite as much as you think is helpful and reassuring to the women.’* **(obstetric physician 1, INT9, the UK)**  Clinical measures to implement a dose recommendation  *‘I’d expect the advice to look something like ‘based on model predictions, the latter changes can be expected in the blood concentration of the medication, meaning that such dose adjustments can be made, but be mindful that the following actions might have to be taken to monitor whether these adjustments are applicable for the individual pregnant woman.’* **(clinical** **pharmacist 2, FG2, Netherlands)**  *‘You need some objective measures (e.g. the number of skin lesions or a blood concentration measure) to check that the MID adjustment in pregnancy also applies to the individual patient.’* (**clinical** **pharmacist 1, FG2, Netherlands)**  *‘You need to outline the symptoms associated with underdosing or overdosing.’* **(clinical pharmacist 5, INT4, Netherlands)**  *‘Risk mitigation measures like antibiotics during chemotherapy should be outlined as part of the dose recommendation, especially if there’s uncertain evidence.’* (**implementation expert, INT5, Netherlands)**  *‘It’s important to outline how underdosing or overdosing can be identified clinically. Which symptoms can you monitor? (…) A matrix where you could tick specific boxes for monitoring could be helpful*.’ **(clinical pharmacist 5, INT4, Netherlands)** |
|  | Stakeholder involvement | MIPF design and impact  *‘Start by interviewing users.’* **(gastroenterologist, INT3, Netherlands)**  *‘(On) co creation, it's important that women feel that they're represented in a way that they can relate.’* **(internist 3,* FG8, Uganda)**  *‘Do all the qualitative research, I guess so patients experiences*.’ (**research physician, INT16, South Africa)**  *‘A lot of the drugs we're using pregnancy are off license for pregnancy and I don't know how you're involving the pharma companies in this as well.’ (***gynaecologist 7, INT6, Vietnam, India, UK)**  Multidisciplinary working committee  *I think the (*involvement of) *ethicists (is) very exciting, I think often when I talk about this area, the questions that I get asked more than the pharmacology and pharmacokinetics is often to do with risk. And of course it's complex because these very unwell women who might need more drugs in pregnancy, the risks may be greater, but the benefits are also greater. So you would accept a greater risk?* **(internist 3,* FG8, Uganda)**  *I think that's very important to have (…) your stakeholders, because it gives people buy in. So for example, once you say that this formulary has been passed through a couple of pregnant women, then it makes other pregnant women more comfortable. So they don't feel like someone has just come and dumped things on them. And I think it's quite important to have those different stakeholders.* **(gynaecologist 11, INT11, Kenya)**  *‘In mostly patriarchal societies, I think it's often (the case) in low and middle income countries where you have mostly the male, the partners to the pregnant women, usually they play an important role.’* **(clinical pharmacologist 6, FG10, Uganda)**  *‘Knowing that respected groups and people have had input, reviewed and just had these kind of discussions, I think it's really reassuring to people.’* **(research physican, INT6, South Africa)**  *‘If it's for targeting things like anti epileptics or biologic agents and all those sorts of things, I guess it's going to have to involve also the relevant disciplines.’ (***gynaecologist 7, INT6, Vietnam, India, UK)** |
|  | Patient information | Rationale for patient information  *‘I think that you must first get a sense of how* (the MIPF*) works and then once it’s clear that it works, you can share information with pregnant women. Because then you will also have more answers to their questions as a professional.’* (**midwife 1, FG7, Netherlands)**  *‘I suppose I'd also be concerned that women might not accept it and, how would I convey this information to somebody that, oh, actually, you know, your sister uses this for her, whatever. But actually you should be using double the amounts and never mind about it. I suppose that would also be a concern is how we would convince women to do it.’* (**clinical pharmacist, INT7, UK)**  *‘If it was put forward to me when I was pregnant, I would have not really been fussed about knowing it was based on a model. (…) But whether it's model based or any other method, it wouldn't really make a difference.’* **(research physician, INT16, South Africa)**  *‘If we have the patient at the heart of everything we do then the data has to be accessible to them. And I agree with patient access for this. If I was a patient, I'd really want to know it.’* (**obstetric physician 1, INT9, UK**)  *‘Pregnant women would definitely want to know about this, especially for drugs with a maternal indication.’* **(gynaecologist 9, INT8, UK)**  Information for various needs  *‘I don't think we should bother them with their dose. The details of the rationale for the change once the change has been endorsed by officials. You can just disseminate the usefulness.’ (***gynaecologist 10, INT10, Cameroon)**  *‘I think that there’s a group of pregnant women that wants to know everything including where I got information from and what it is based on. It would be useful to have background information for this group***.’ (general practitioner 1, FG3, Netherlands)**  *‘I think in our context we're dealing with broadly two groups of patients. We're dealing with patients within the public sector and patients we see within the private sector, which I think is really different because sometimes private sector patients are more in tune with what's happening. (…) I think if they get a different dose to what's being put out there they will question it (…). Within the public sector I don't think we have much patients like that. (…) I think it would be good to give them enough information such as this dose is different from the normal dose*. (…) *Information like that should be fed through the clinician.’* (**clinical pharmacologist, INT16, South Africa**) |
|  | Clinical interpretation | *‘I would recommend that a gastroenterologist be involved in assessing the intestinal bowel disease medication* (for an individual patient) *because it’s not a black and white matter, clinical remission must be considered alongside blood concentrations.*’ **(gastroenterologist, INT3, Netherlands)** |
| Access |  | Up-to date centralized information  *‘It’s difficult to keep up to date on all the existing knowledge on (medication in pregnancy).****’* (gastroenterologist, INT3, Netherlands)**  *‘I would like to see all the information (on medication in pregnancy) bundled in one place.’* **(community pharmacist 1, FG5, Netherlands)**  *‘If you've got a patient in front of you and you need the information, then actually you need it from a single source.’* (**obstetric physician 1, INT9, UK)**  (Hosting) website  *‘Making* (the MIPF) *available on a website (…) would fit the daily routine of general practitioners well.*’ (**general practitioner 1, FG3, Netherlands)**    ‘*A standalone website would give more face recognition to the formulary*.’ (**clinical** **pharmacist 5, INT4, Netherlands)**  *‘I would see it more like a stand-alone website.*’ **(community pharmacist 2, FG5, Netherlands)**  *‘In my opinion a pregnancyformulary.nl website would make sense.’* **(community pharmacist 3, FG5, Netherlands)**  *‘Don’t create yet other sources of information. (…). I would prefer to use something that we already use.’* (**gynaecologist 5, FG6, Netherlands)**  *‘I would plug into existing portals or websites that are already widely used and to not reinvent the wheel.’* **(general practitioner 3, FG3, Netherlands)**  *‘There should be a link* (to the MIPF) *on the Dutch formulary, which is pretty much (…) used by all doctors***. (general practitioner 1, FG3, Netherlands)**  *‘The Dutch TIS is really our most used and trusted reference.’* **(midwife 1, FG7, Netherlands)**  Mobile application  *‘I think that you will have to create an app. At least this would make (dose recommendations on the MIPF) easy to implement for anaesthetists because we tend to quickly look up information on our phone.’* (**anaesthetist 3, INT2, Netherlands)**  Talking about an applicaton providing individualized advice based on blood concentrations of a medication: ‘*Doctors thought it was too much of a hassle to enter data in the app, it cost too much time.’* This was the main reason for the application not to be further developed after user testing. (**neurologist, INT1, Netherlands)**  ‘I*t should be accessible on the phone. And then if there was a way you could have a downloadable version*.’ **(clinical pharmacologist 6, FG10, Uganda)**  *‘Then it’s more about getting in the hands of clinician. It's like having a really good app that's really easy to use and that you can push out on social media.’* (**obstetric physician 2, INT12, UK)**  Navigation  *‘A website must be user-friendly. It must be easy to collect information, (…) three clicks is already too much.’* **(community pharmacist 1, FG5, Netherlands)**  **‘***You must clearly distinguish between information for patients and for HCPs and employees****’*. (community pharmacist 2, FG5, Netherlands)**  *‘I think online definitely, but I think it would be quite useful if it was layered in that you could have as much information as you as you needed in different layers.’* (**clinical pharmacist, INT7, UK)**  Patient information  *‘I like the videos because, even for someone who's illiterate (…) these videos can be translated to many of the local languages.’* **(clinical pharmacist, FG8, Uganda)**  *‘Most uneducated women, even they understand that, for example, taking alcohol (…) or smoking when you're pregnant isn't advisable. (…) I think even drawing from parallels that we know of in the community, just simply using an example that is almost known worldwide that when you're pregnant, (…), then you begin to show them that some of these drugs might have similar effects.’* **(clinical pharmacist, FG8, Uganda)**  *In my view, patients have the best knowledge of their body and are the main person responsible for their treatment so you should share information with them. How openly you do this depends on the indication. For instance, for antipsychotics, given the risk of raising concerns when no interpretation is given, more personal guidance is needed, for example a conversation with a doctor or a pharmacist. (…) I wonder whether a website is the best format.’* **(clinical pharmacist 5, INT4, Netherlands)**  ‘*It would help to have a demonstration model to show the concept to pregnant women*.’ (**gynaecologist 9, INT8, UK**)  *‘I think both* (online information and the clinician as sources) *to be honest. I think It's always good having that initial conversation in a consultation. But then obviously that's a hell of a lot of information for them to absorb in one go. And I always think having another resource for them to sit and properly look at is also really useful. And also, it just shows it's not just us talking about this sort of abstract concept, there’s actually written resource that they can look at themselves.’* (**obstetric physician 3, INT13, UK)**  *I think the UK model with two separate kinds of information sources* (UKTIS website for HCPs and Bumps interface for patients*). I think that works well.* (**obstetric physician 3, INT13, UK)**  *‘I think the other thing that's helpful, particularly with regards to risk is to give people a pictorial representation.’* **(midwife, INT13, UK)** |
| Sustainability |  | ‘*Funding is crucial and the biggest hurdle to overcome. Sustainable finance needs to be obtained so that the MIPF can stand on its own feet.’* **(implementation expert, INT5, Netherlands)**  ‘*Setting up a stand-alone entity (…) will help with the obtention of name recognition and with fundraising*.’ **(clinical pharmacist 5, INT4, Netherlands)** |
