# Supplementary material for: Perceived barriers and facilitators for model-informed dosing in pregnancy: a qualitative study across healthcare practitioners and pregnant women
Source: BMC Med. 2024 Jun 18;22:248. doi: 10.1186/s12916-024-03450-8 (PMC11184760; doi:10.1186/s12916-024-03450-8)
Supplement: Supplementary file 6 — Additional file 6. Quotes from pregnant women. [file 12916_2024_3450_MOESM6_ESM.docx]

**Additional file 6 – Quotes from pregnant women**

All pregnant women that participated in focus groups or interviews resided in the Netherlands. Quotes that were originally in Dutch were translated in English.

**Quotes from pregnant women on their perceived barriers and facilitators for the implementation of a model-informed pregnancy formulary -** FG: focus group; HCP: healthcare practitioner, INT: interview; TIS: Teratology Information Service.

1. INNOVATION

| Relevance | Information gap | Medication use and fetal safety  *‘Medication leaflets are very general and nobody is willing to take a risk. You will not get a definite answer* (on whether the medication can safely be used*).* (…) *I think nobody can tell you with 100% certainty. There is always a caveat about the fact that we don’t really know if there are any cases* (of teratogenicity).’ (pregnant woman 5, with bipolar disorder, FG11)  *‘Doctor Google will tell you that you will get horrible things* (from using medication in pregnancy*). (…) It’s also problematic that* (the relevant information) *is not always known by pharmacists.’* (pregnant woman 9, doctor, FG12)  *‘What do medicines like painkillers do on the life of the unborn child?’* (pregnant woman 3, with anxiety disorder, nurse, FG11)  *‘I pro-actively looked for information at that time* (during her pregnancy), *from Google searches to the Dutch formulary. But none of these sources provided clarity* (on which medication to use) *other than advising caution during pregnancy and telling me to discuss my medication choice with my carer.’* (pregnant woman 5, with bipolar disorder, FG11)  *‘I received incorrect medication advice (*from my general practitioner) *before my pregnancy which led to me feeling unwell, which in turn influenced my decision of whether or not I wanted to undergo a pregnancy.’* (pregnant woman 3, with anxiety disorder, nurse, FG11)  *‘When you ask a doctor he will give you information. You ask your mom she will give you information. Go to the Internet, you can see other information so there is a lot of information.’* (pregnant woman 10, INT14)  *‘You have to trust the psychiatrist’s advice and otherwise there is not much information to be found (…) so there is room for improvement.*’ (pregnant woman 5, with bipolar disorder, FG11)  *‘What I needed was consensus from the medical community (with regards to whether medication could safely be used during pregnancy).*’ (pregnant woman 3, with anxiety disorder, nurse, FG11)  Medication doses  *‘Everything that I have read* (on doses of anti-epileptics during pregnancy) *is very, very superficial and feels like it doesn’t help me at all.’* (pregnant woman with epilepsy, INT15)  *‘I feel that there is still little certainty about both (*appropriate*) types of medication and medication doses* (in pregnancy*).’* (pregnant woman 3, with anxiety disorder, nurse, FG11)  Breastfeeding  ‘*What I struggled with most, was whether I should breastfeed the baby.’* (pregnant woman 11, with epilepsy, INT15)  *‘Shortly after my delivery, (…) I needed a lot of valium and morphine. (…) There was quite a lot of discussion between the gynaecologists and pharmacists on whether this influenced the breastfeeding. So I think it’s very relevant.’* (pregnant woman 6, doctor, FG12) |
| --- | --- | --- |
|  | Clinical relevance | Maternal and fetal treatment  *‘It’s very important to know the extent to which the medication is going to affect the baby and how much it affects the mother as well.’* **(pregnant woman 10, INT14)**  Impact of suboptimal maternal treatment  *‘If your medication doesn’t work as intended* (in pregnancy), *this means that you’ll suffer from your chronic condition, which may be challenging*.’ **(pregnant woman 11, with epilepsy, INT15)**  Impact of uncertainty  *‘**The uncertainty around my medication (…) and what was best for me and for my baby and whose advice I could believe gave me a lot of stress. It was a vicious circle.’* **(pregnant woman 3, with anxiety disorder, nurse, FG11)**  *‘I was very scared to be forced to switch to an alternative* drug.*’* **(pregnant woman 5, with bipolar disorder, FG11)**  Reducing the need for participation of pregnant women in clinical studies  *‘This is an elegant way to collect all the data, that is, not as part of a clinical study but from behind a desk.’* **(pregnant woman 5, with bipolar disorder, FG11)** |
| Feasibility |  | Fetal safety  *‘What are the ultimate effects of the small amount of drugs that arrives to the baby? That seems to be a challenging part, isn’t it? Because how do you determine what is acceptable?’* **(pregnant woman 10, INT14)** |
| Complexity |  | *'To be honest, I felt like it's kind of difficult to understand.'* (**pregnant woman 10, INT14)** |

2 USERS

| Awareness |  | Of HCPs  *‘Doctors’ awareness of* (the link between physiological changes in pregnancy and the need for dosing adjustments in that period*) must grow.’* (pregnant woman 11, with epilepsy, INT15)  *‘**I think that first doctors should realize how big an impact* (a poorly treated chronic condition) *has on the pregnancy and on the associated happiness. (…) The awareness* *must be there first. Otherwise they* *won’t invest energy in gaining a better understanding of this. But this often has to do with the attitude of a given doctor so that’s difficult*.*’* (pregnant woman 11, with epilepsy, INT15) |
| --- | --- | --- |
| Knowledge |  | Physiological changes in pregnancy  *‘I felt guilty because I always thought (…) that* (the anti-epileptic medication dose) *was mostly influenced by hormones (…). And then I was told that actually my pregnancy affected the kidneys too. Why had I not found this before? I thought, how stupid, you should know that you get more blood (…) and I felt guilty.’* **(pregnant woman 11, with epilepsy, INT15)**  Medication doses  *‘They drew blood just now and we increased the medication by 100 mg last time. If it still was going badly (with the epilepsy insults)/ I could add another 150. Last time my blood concentration went from 1.8 to 1.9 and for me the medication works when it is at 7 so I wonder what such an increase does.’* **(pregnant woman 11, with epilepsy, INT15)** |
| Attitude | Reference framework | Fetal safety as a key concern for pregnant women  *‘What I found most important was: what risks is my child exposed to?’* **(pregnant woman 11, with epilepsy, INT15)**  Fetal safety as a key priority for HCPs  ‘*Remarks from doctors like ‘but you want a child, right? Then that’s just how it is for you. I think that it’s really not OK but it happens. Or general practitioners that have told me ‘it’s just for nine months.’* **(pregnant woman 11, with epilepsy, INT15)**  *‘I could see that the psychiatrist was very relaxed and open in this regard* (her giving priority to preventing manic or depressive episodes during her pregnancy*). He just says, of course, it is very important.’* **(pregnant woman 5, with bipolar disorder, FG12)**  Maternal health  *‘A stable mother is the most important thing and the rest will follow suit* ***.***’ **(pregnant woman 5, with bipolar disorder, FG12)**  *‘You want to be happy during your pregnancy. Of course you are focused on your child, but it isn’t pleasant to not feel like yourself twice for nine months in your life* (in reference to the increased frequency of epilepsy insults that she undergoes in her pregnancy*).’*  **(pregnant woman 11, with epilepsy, INT15)**  *‘For me it* (maternal health) *is more of a consideration. I have been stable for ten years. Because I know that I don’t want to undergo a manic episode or a depression (…), I try to do everything to prevent this.’* **(pregnant woman 5, with bipolar disorder, FG11)**  *‘If you were only looking at my blood concentration level* (of the medication), *you would give other advice. But you may also ask: in what other aspects of* (her) *life can I make changes to improve my condition* (during pregnancy)?*’* **(pregnant woman 11, with epilepsy, INT15)**  Models  *‘There is so much that is done by models these days that it’s funny that this is not yet the case in (…) the medical world.*’ (**pregnant woman 1, FG11)** |
|  | Information needs | Fetal safety  Talking about her hesitancy to use antacid medication to treat the reflux symptoms she experienced during her pregnancy: *‘It’s very weird but (…) you are way more preoccupied with your child and what is good or bad (for her) and you are a lot more careful than with yourself. (…). This was not at all conscious for me but I was way more concerned by* (medication*) safety than I normally would be.’* **(pregnant woman 8, doctor, FG12)**  *‘Your emotions seem to take over your rational thinking somewhat so I recognize this. (… ) You know that you can take it but you still hesitate and check all kinds of sources twice.’* **(pregnant woman 9, doctor, FG12)**  *‘I have never admitted a pregnant woman to hospital because she had symptoms after taking too many (*antacids*) so I am fully aware that it is not harmful and still, there was a moment where I thought, I just have to be sure because it is about your own child, which of course is really stupid. But this is how it goes.’* **(pregnant woman 9, doctor, FG12)**  Medication doses  *‘**As a patient, I want to know when my medication dose must be increased.’* **(pregnant woman 11, with epilepsy, INT15)**  *‘Whether I want to be informed about the dose depends on the expected side effects, for example if more side effects are expected from using 1000 mg of paracetamol than from using 500 mg (…), it may be useful to have more information about the dose.’* **(pregnant woman 3, with anxiety disorder, nurse, FG11)**  ‘(The dose) *is something for the doctors.*’ **(pregnant woman 10, INT14)**  *‘They were talking about maybe increasing* (the dose of) *my medication towards the end* (of the pregnancy). *(…) This is something I want to know. (…) If* (increasing the dose) *can help maintain some stability so that being a healthy mother is prioritized, I would like to be aware that this option exists.’* **(pregnant woman 5, with bipolar disorder, FG11)**  *‘I can imagine that you would feel more confident if you had information about why* (HCPs) *are almost certain that this is the right dose*.’ (**pregnant woman 2, FG11)**  *‘I* *would like to know everything*. (…) *But maybe this has to do with my medical background*.’ **(pregnant woman 3, with anxiety disorder, nurse, FG11)**  ***‘****If* *the dose of medication that is normally used has to be increased in pregnancy, it would be nice if the doctor can say that recent research has shown that the antibiotic* (in the case of a bladder infection*) is not effective at all if you are pregnant. You must take more because you pee so much. As a patient I would find this information very plausible and would like to be told*.’ **(pregnant woman 6, doctor, FG12)**  *‘I didn’t look for additional evidence. (…) I wasn’t going to need any exotic medication so I didn’t try to check everything with 100% certainty because I knew before starting* (the medication*) that it’s always* allowed (during pregnancy). *But it might have been different if I had been needed an uncommon antibiotic, then I might have looked further.‘* (**pregnant woman 6, doctor, FG11)**  Model-informed dosing  *‘I think if he* (the doctor) *explains this shortly, it will be enough, because it might not be easy to understand (…).. It will make things even more complicated.’* **(pregnant woman 10, INT14)**  About whether they would like to have access to a resource on model-informed dosing in pregnancy: *‘Yes, I am in favor of transparency so how do you get your information and what can you get out of it, how can you interpret this?’* (**pregnant woman 3, with anxiety disorder, nurse, FG11)** |
|  | Willingness to change | Of pregnant women  *‘If you’re going to advise higher doses (…), as a pregnant woman, I would think that more isn’t always better. In addition, if I play devil’s advocate, then if my bladder infection disappeared with 3 times 500 amoxicillin all those years, why should I suddenly used 3 times 1000?*’ (**pregnant woman 8, doctor, FG12)**  Of HCPs  *If this* (attitude of HCPs not prioritizing an appropriate treatment of maternal disease in pregnancy*) remains, then you can make a model, but if someone thinks it* (the effectiveness of maternal treatments) *is not that important, they won’t use it*.’ **(pregnant woman 11, with epilepsy, INT15)** |
|  | Shared decision-making | Roles and responsibilities  *‘The doctor is not responsible for everything. The patient is also partly accountable for her own health.’* **(pregnant woman 11, with epilepsy, INT15)**  ‘(Decisions around medication in pregnancy) *are your own responsibility* (as a pregnant woman) *until you discuss those with the physician. (…) Once you share a symptom with the doctor and the doctor knows that you are pregnant and tells you ‘you can use this’ (…), then the responsibility lies with him.’* **(pregnant woman 6, doctor, FG12)**  *‘I have the feeling that I can go into discussion, so there is a shared decision in this regard. (…) Ultimately you* (the pregnant woman*) are always the one that makes the choice. But they* (your HCPs) *have the knowledge so they give you the advice. In general I will also follow this advice but I will go into discussion if I need to because I want to understand why you do something.’* **(pregnant woman 11, with epilepsy, INT15)**  *‘I think that you can consider the pros and cons together (…). Ultimately the patient decides whether or not she will take* (the medication) *but the doctor is responsible for safety when he or she prescribes.’* **(pregnant woman 9, doctor, FG12)**  *‘I think that it’s the responsibility of the physician to share the information and the options (…) but that the choice lies with the patient. Of course you have hormones as a pregnant woman but you can make decisions when you are well informed.’* (**pregnant woman 7, doctor, FG12)**  *‘I actually did not want to have that choice* (about whether to take medication). *I preferred to have someone, a doctor (…) that I would really trust, that could tell me that it would be fine. It’s a very difficult decision to make because you are deciding about a child that isn’t born yet. I wanted to be a good mother but how to do this.’* **(pregnant woman 3, with anxiety disorder, nurse, FG11)**  *‘The responsibility* (of decisions about medication in pregnancy*) is completely mine.’* **(pregnant woman 5, with bipolar disorder, FG11)**  Process  *‘I feel that if you want to deliver good care, so if you really want (*the patient) *to* (make decisions) *together with the doctor, then the patient should have access to* (some information). *They don’t need to fully understand the model but simply explain that you have more blood when you are pregnant.’* **(pregnant woman 11, with epilepsy, INT15)**  *‘This* (the information provided by the psychiatrist) *gives me confidence that while the child may be less alert for 24 hours, (…) this is acceptable. This is something that I cannot really assess myself so this gave me confidence that this was OK.’* **(pregnant woman 5, with bipolar disorder, FG11)**  It gives her trust ‘*that* *there is a balanced assessment between the potentially harmful effects* (on the baby) *versus how much* (the medication) *helps you, that* (the latter) *is also very important, and that you can make the choice yourself. This choice does not have to be made for you. If you exactly know* (the pros and cons), *then I think that everyone would be able to make this choice.’* **(pregnant woman 1, FG11)** |
| Behaviour | Information search and sources | *‘Between my pregnancies I spent a lot of time researching medication doses to get it right the next pregnancy.* (My medication regimen) *did not work during my* (first) *pregnancy so I have really been applying myself to this. I receive very good clinical guidance but I’m also very active myself searching for information on PubMed.’* **(pregnant woman 11, with epilepsy, INT15)**  *‘My information came from many different sources, that is a combination of online sources, different doctors as well as women that had been pregnant with my condition.’* **(pregnant woman 3, with anxiety disorder, nurse, FG11)**    *‘I looked for information on ibuprofen on the medication leaflet.’* (**pregnant woman 2, with migraine, FG11)**  *‘Every day I read on the Internet what I should and shouldn’t take (…)* *I just type what I want like ‘is omega-3 good during pregnancy’ (…). And* (look) *at the first 2-3 options I get. I try to pick the one with some kind of medical logo or something.’* **(pregnant woman 10, INT14)**  *‘I will compare* (the doctor’s) *answers with what I read and I can discuss it with him as well.’* **(pregnant woman 10, INT14)**  *‘When you Google information, you primarily find information from women’s forums and stories from pregnant women. (…) I find that relevant information is not easy to find for patients.’* **(pregnant woman 6, doctor, FG12)**  ‘*I looked up* (information about medicines) *on websites before I went to the general practitioner*.’ **(pregnant woman 4, FG11)** |
|  | Medication use | ‘*I can manage, including without medication. It is just more difficult but it is feasible to go through an attack without medication. Because it only lasts a few hours after all.*’ (**pregnant woman 2, with migraine, FG11)**  *‘When I think about it, I thought about whether I really needed to* take (a given medication) *much more. I thought: can I not skip it for once and just go to bed earlier and then the headache will be gone than I would now, where I would just continue doing what I am doing and take two paracetamols.’* **(pregnant woman 9, doctor, FG12)**  *‘You’d rather be sure so if it is not absolutely needed,* *then you’d rather not take it.***’ (pregnant woman 8, doctor, FG12)** |

3 SOCIAL AND ORGANISATIONAL FACTORS

| Culture |  | *‘I find this very interesting because medication in pregnancy (…) has become a real topic of discussion. Things are not self-evident anymore, and what you used to do because your midwife or doctor told you so, that is now undergoing some interesting developments.’* (pregnant woman 1, FG11) |
| --- | --- | --- |

4 IMPLEMENTATION

| Awareness raising and education |  | Of HCPs  *‘Doctors’ awareness of* (the need for pregnancy-adjusted doses*) must grow. (…) So this should actually become knowledge that is part of their training.’* (pregnant woman 11, with epilepsy, INT15) |
| --- | --- | --- |
| Access | Website | On physiological changes in pregnancy: ‘y*ou would put* (this information) *on a website that is specifically dedicated to this. (…). A doctor could tell you to have a look at this so that you have this background knowledge.’* **(pregnant woman 11, with epilepsy, INT15)**  *‘I don’t think I would (*consult a website*). You sometimes get curious about specific things that you would like to research, but in general, no, I don't think it's necessary.*' **(pregnant woman 10, INT14)**  *‘I would very much like to have* (a website with information on model-informed dosing in pregnancy). (…). *The doctor may not have time to give you a detailed explanation; this would of course require a lot of time. If he could then tell you about this place where it* (this information*) is clearly explained I think it would be nice.’* **(pregnant woman 1, FG11)**    ‘The information on medication use in pregnancy can be on a website but ‘*then you must make sure that* (answers to the question) ‘*can this harm my baby’ be placed on the first page of Google* *results.* *(…) Until this is the case* (forums like) *viva.nl will always win from* (medical websites like) *gynaecoloog.nl.*’ **(pregnant woman 6, doctor, FG12)** |
|  | HCP | *‘On the one hand, I like to look up information myself. But how do you ensure that you are not responsible for sharing an excess of information? How do you make sure that this information is understandable for the lay person (…)? Do you rely on the doctor to ensure that the information doesn’t start living a life of its own? I think it really depends on how you communicate the information.’* **(pregnant woman 5, with bipolar disorder, FG11)**  *‘The doctor could tell you: look at this* (website*). And then she could have a conversation with the patient about how they can both manage the pregnancy.’* **(pregnant woman 11, with epilepsy, INT15)**  *‘Bad experiences come up online more quickly than the good experiences, so I think that a doctor and a midwife have a large role to play still* (in advising women on medication use in pregnancy).’ **(pregnant woman 6, doctor, FG12)** |
| Usability | MIPF content | Fetal exposure  *‘I would like to know* (about the predicted fetal exposure). *Because right now I have to trust a doctor but I don’t know anything about whether the medication goes through the placenta.’* **(pregnant woman 5, with bipolar disorder, FG11)**  Physiological changes in pregnancy  ‘*If you want to deliver good care (…) patients must also have access to* (some information). *They don’t need to understand the entire model. Simply explain to them: you have more blood when you are pregnant (…) or the kidneys work differently. This can be pretty easily explained so people understand this better.’* (**pregnant woman 10, with epilepsy, INT15)** |
|  | Language | ‘(Information on doses in pregnancy) *must be made freely accessible. A patient must be able to read the information and this information must be written in a language that can be understood by her.’* **(pregnant woman 11, with epilepsy, INT15)** |
